# The IFNγ‐CIITA‐MHC II axis modulates melanoma cell susceptibility to NK‐cell‐mediated cytotoxicity

**DOI:** 10.1002/1878-0261.70133

**Published:** 2025-10-13

**Authors:** Lena C. M. Krause, Rixa‐Mareike Köhn, Christian Ickes, Julia Lenger, Jonas Fischer, Sabrina Cappello, Ivan Bogeski

**Affiliations:** ^1^ Molecular Physiology, Institute for Cardiovascular Physiology, University Medical Centre University of Göttingen Germany

**Keywords:** IFNγ, immunotherapy, melanoma, NK cells

## Abstract

Melanoma, the deadliest form of skin cancer, poses a significant challenge due to its genetic heterogeneity and high metastatic potential. While cytotoxic T cell (CTL)‐based immunotherapies have made remarkable progress in recent years, the therapeutic potential of natural killer‐(NK) cells is increasingly recognized. However, resistance mechanisms to both CTL‐ and NK‐cell‐mediated immunotherapies hinder effective treatment. To evaluate the exclusive role of NK‐cells in anti‐melanoma immunity, we performed *2D* and *3D* co‐culture‐based cytotoxicity assays under varying conditions. Our findings revealed a protective phenotype in melanoma cells following prolonged exposure to primary NK‐cells. By combining experimental data with bioinformatic analyses, we identified key genes and pathways involved in melanoma cell adaptation to NK‐cell‐mediated killing (NKmK). We found that cytokines such as IFNγ play a major role in suppressing NKmK with MHC II surface expression being a critical factor. Targeting the master regulator CIITA, which governs MHC II expression and is affected by IFNγ, significantly reduced melanoma cell resistance to NKmK. This study provides potential strategies to overcome resistance to NK‐cell‐based immunotherapies and offers novel insights into melanoma immune escape mechanisms.

## Graphical abstract

Natural killer (NK) cells play a central role in anti‐melanoma immunity. However, melanoma cells adapt during co‐culture by upregulating CIITA and MHC II in response to interferon gamma (IFNγ), thereby evading NK‐cell lysis. Blocking IFNγ signaling or treatment with dimethyl fumarate/simvastatin counteracts this immune escape and maintains NK‐cell cytotoxicity.

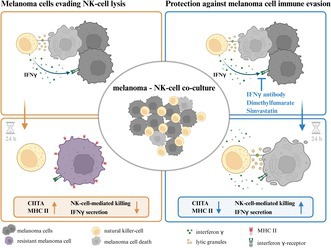

**Abbreviations**
CEACAM1: carcinoembryonic antigen‐related cell adhesion molecule 1
CIITA: MHC class II transactivator
CML: chronic myelogenous leukemia
CRTAM: cytotoxic and regulatory T cell molecule
CTL: cytotoxic T lymphocyte
DC: dendritic cell
DMF: dimethyl fumarate
HLA: human leukocyte antigen
IFNGR: interferon gamma receptor
IFNγ: interferon gamma
IL‐2: interleukin‐2
LAG‐3: lymphocyte activation gene 3
LGALS9: galectin‐9
MHC: major histocompatibility complex
NK‐cells: natural killer cells
NKmK: NK‐cell‐mediated killing
PBMC: peripheral blood mononuclear cell
scRNAseq: single‐cell RNA sequencing
siRNA: small interfering RNA
ULBP2/3: UL16 binding protein 2 and 3

## 1 Introduction

Melanoma, as one of the most aggressive and deadliest skin cancers [1], is hard to cure once it has spread to distant organs [2, 3]. In recent years, targeted therapies, frequently aiming for the RAS–RAF–MEK–ERK signaling cascade, as well as immunotherapy approaches, showed great advances in disease outcome. Nevertheless, drug resistance and low response rates present a serious problem [4], highlighting the need for improved therapeutic strategies and a deeper understanding of melanoma evasion mechanisms.

Natural killer‐(NK) cells are at the first line of defense against tumor cells. Their repertoire of germ‐line encoded receptors provides a fine balance between activating and inhibitory signals that control their cytotoxic potential [5]. Similar to T cells, NK‐cells exert immunomodulatory functions through the secretion of cytokines [5, 6] and cytotoxic activity through several mechanisms, such as the release of lytic granules containing mainly perforin and granzyme B [7]. In the context of cancer, NK‐cell cytotoxicity correlates with a lower risk of developing tumors and with spontaneous cancer regression [8]. Also, infiltrating NK‐cells can be a positive prognostic factor for patients with melanoma and other cancers [9, 10]. Melanoma is highly immunogenic [11] and often exhibits features that enable NK‐cell recognition and lysis. For instance, due to frequent loss of MHC I molecules in melanoma cells [12], NK‐cell recognition and melanoma cell lysis is high *in vitro* [13]. However, despite of promising clinical trials [14], *in vivo* melanoma often exerts evasion mechanisms from NK‐cell lysis, posing difficulties for the clinical use of NK‐cells in melanoma therapy [13].

Previous studies exploring melanoma cell–NK‐cell interactions revealed the development of a protective phenotype of melanoma cells, showing less susceptibility to NK‐cell‐mediated killing (NKmK), which was mediated through IFNγ signaling [15, 16, 17, 18, 19, 20]. This protective or ‘resistant’ melanoma phenotype was accompanied by an increased MHC I expression, which was proposed to be linear to the number of NK‐cells that the melanoma cells were co‐cultured with [17]. Moreover, the elevation in MHC I was found to be protective against NK‐cell‐mediated lysis [20]. To this end, it should be noted that the role of MHC I in NK‐cell‐mediated cytotoxicity has already been extensively studied [21, 22, 23, 24]. Notably, the role of MHC I expression is still not fully understood. For example, MHC I did not correlate to NKmK in nontreated melanoma cells [25]. Additional studies demonstrated that IFNγ‐dependent resistance formation is independent from HLA I antigen expression in melanoma but also in K562 chronic myelogenous leukemia (CML) cells [15, 16]. This clearly demonstrates the need to better understand the development and the molecular mechanisms involved in determining melanoma resistance formation, that is, melanoma cell sensitivity to NK‐cell‐based therapies.

In this study, we demonstrate the existence of adaptive mechanisms in genetically heterogeneous melanoma cell lines using *2D* and *3D* co‐culture‐based model systems. Notably, even short‐term co‐culture with NK‐cells induced a protective phenotype in both primary and metastatic melanoma cells. By analyzing single‐cell RNA sequencing (scRNA‐seq) patient datasets, we identified IFNγ as a predicted key interactor between melanoma and NK‐cells. Our experimental data confirmed IFNγ release during co‐culture and revealed a strong correlation between IFNγ concentration and melanoma cell susceptibility to NKmK. Furthermore, we found that IFNγ is a central driver of melanoma sensitivity to NKmK. RNA‐sequencing analysis from previous studies showed that IFNγ regulates a broad set of genes, with MHC class II molecules being among the most strongly affected. We validated these findings by exposing melanoma cells to IFNγ and after NK‐cell co‐culture. Finally, we demonstrated that pharmacological inhibition of the IFNγ‐induced CIITA pathway using dimethyl fumarate (DMF), an FDA‐approved drug for psoriasis and multiple sclerosis [26, 27, 28], effectively interferes with the adaptation mechanisms, rescues resistance formation, and restores MHC II upregulation in melanoma cells. Our data suggest that CIITA plays a crucial role in melanoma resistance formation, while MHC II expression may serve as a potential resistance biomarker and therapeutic target. In summary, our findings reveal novel targets for overcoming melanoma resistance and may contribute to the improvement of NK‐cell‐based immunotherapies.

## 2 Materials and methods

### 2.1 Cell culture and reagents

#### 2.1.1 NK‐cells

For primary NK‐cell isolation, leukocyte reduction system chambers were provided by the local blood bank of the Institute of Transfusion Medicine at the University Medical Centre Göttingen (UMG Ethics approval 2/3/18). Peripheral blood mononuclear cells (PBMCs) were isolated from healthy, nonsmoking donors by density gradient centrifugation using LeucoSep tubes (50 mL, Greiner Bio‐One: Frickenhausen, Germany, #277290) and Lymphocyte Separation Medium 1077 (PromoCell: Heidelberg, Germany, #C‐44010). Primary NK‐cells purification was performed from fresh PBMCs using the NK‐cell Isolation Kit from MACS Miltenyi Biotec (Bergisch Gladbach, Germany, #130‐092‐657) following the manufacturer's protocol. Primary NK‐cells were maintained in AIMV medium (ThermoFisher Scientific: Darmstadt, Germany, #12055‐091) supplemented with 10% fetal calf serum (FCS, Sigma‐Aldrich: Taufkirchen, Germany, #A9418). NK‐cells were activated by adding 0.05 mg·mL^−1^ IL‐2 (Hycultec: Beutelsbach, Germany, #Hy‐P7037) 24 h prior to following procedures. This study was conducted in accordance with the principles outlined in the Declaration of Helsinki. Written informed consent was obtained from all participants prior to their inclusion in the study.

#### 2.1.2 Target cells

The human leukemia cell line K562 (#CCL‐243, RRID: CVCL_0004) was obtained from the American Type Culture Collection (ATCC) and was used as a positive control for NK‐cell killing. The K562 cells were cultured in RPMI1640 medium (Thermo Fisher Scientific, #21875‐034) supplemented with 10% FCS. The melanoma cell lines 1205Lu (RRID: CVCL_5239), WM793 (RRID: CVCL_8787), WM1366 (RRID: CVCL_6789), and WM3734 (CVCL_6800) were kindly provided by Prof Meenhard Herlyn (Wistar Institute, Philadelphia, PA, USA). All melanoma cells were maintained in melanoma growth medium TU2%, consisting of 80% MCDB153‐Basal medium (Biochrom AG: Berlin, Germany, #F 8105), 20% L15 Leibowitz Medium (PromoCell, #C24300), 2% FCS, 1–0.68 mmol·L^−1^ CaCl_2_ (Sigma‐Aldrich, #21115), and 200 mmol·L^−1^ l‐Glutamine (Sigma‐Aldrich, #G7513100ML). Melanoma cell lines were authenticated by short tandem repeat (STR) profiling within the last 3 years. All cell lines were regularly tested for *Mycoplasma* contamination by using the PCR Mycoplasma Test Kit I/C (PromoCell, #PK‐CA911024), confirming that all experiments were performed with *Mycoplasma*‐free cells. Cell numbers were determined using Countess III FL (Thermo Fisher Scientific) and 0.4% trypan blue (Life Technologies).

#### 2.1.3 Co‐culture of melanoma and primary NK‐cells

For co‐culture of melanoma and primary NK‐cells, one million melanoma cells were seeded in a T25 cell culture flask 24 h prior to co‐culture in TU2% melanoma medium. Primary NK‐cells were counted and added in a 1 : 1 ratio to the melanoma cells. Co‐culture was maintained for 24 h, and NK‐cells were removed by taking off the supernatant. Melanoma cells were washed a minimum of three times using 5 mL PBS. Microscopic control ensured that NK‐cells were washed off the melanoma cells before further analysis. For recovery experiments, melanoma cells were cultured with fresh TU2% after NK‐cell removal for 24, 48, or 72 h. Untreated melanoma cells at the same passage and in equivalent culture conditions were used as a control for all experiments and analysis.

### 2.2 Melanoma drug treatment

To interfere with melanoma resistance formation, we applied several compounds to the melanoma cell culture. For IFNγ treatment, melanoma cells were seeded as described before (chapter 4.1.3) and 350 ng·mL^−1^ (if not indicated differently) Recombinant Human IFNγ (carrier‐free, BioLegend: Amsterdam, Netherlands, #570202) was added. Analyses were performed 24 h after treatment initiation.

Either alone or in combination with IFNγ, we applied DMF (75 μm, Sigma, #242926‐25G) or simvastatin (10 μm, Sigma, #S6196) to melanoma cells. IFNγ blocking was performed using an IFNγ‐targeting monoclonal antibody (10 μg·mL^−1^, BioLegend, #506532), or an isotype control (BioLegend, #400166), added during the co‐culture with NK‐cells. When using antibodies during co‐culture, primary NK‐cells were treated with an Fc block (BioLegend, #422302) to inhibit the interaction of CD16 with the Fc part of the antibodies. LAG‐3 blocking was performed during cytotoxicity assays using an anti‐LAG‐3 blocking antibody (17B4, AdipoGen: Füllinsdorf, Switzerland, #AG‐20B‐0012).

### 2.3 Real‐time cytotoxicity assay

Cytotoxicity of primary NK‐cells was analyzed using a real‐time cytotoxicity assay as described previously by Kummerow et al. [29]. Target cells (melanoma or K562) were stained with 0.5 μmol·L^−1^ calcein‐AM (Life Technologies, #C1430) for 15 min and seeded into a black, clear‐bottom 96‐well plate (Corning: Kaiserslautern, Germany, #3904; Greiner Bio‐One, #655090) in duplicates or triplicates. Cell death due to NK‐cell‐mediated killing (NKmK) was measured by the decrease of fluorescent signal over time (Ex.: 485 nm; Em.: 535 nm), recorded with a CLARIOstar plate reader (BMG LABTECH: Ortenberg, Germany), set to bottom‐readout. Fluorescence was measured every 10 min. Triton X‐100 lysed target cells were used as positive controls (PC) whereas living target cells alone were negative controls (NC) for cell death.

The following index I corrects fluorescence differences at baseline (*t* = 0).

$$I:I_{t}=0=Y_{t=0}/{\mathrm{NC}}_{t=0}$$

$$\text{NKmK}=\frac{Y-\left(\mathrm{NC}\times I\right)}{I\times \left(\mathrm{PC}-\mathrm{NC}\right)}\times 100$$

### 2.4 Melanoma spheroid killing

Melanoma spheroids were embedded as described previously [30]. Shortly, 5000 cells were seeded in an ultra‐low attachment plate (Corning, #7007) in 100 μL TU2%. The cells were allowed to form spheroids for 3 days before adding IFNγ‐treatment (350 ng·mL^−1^). After an additional 24 h, spheroids were embedded into a collagen matrix in a 48‐well plate. Spheroids were harvested in 100 μL of 2 mg·mL^−1^ bovine collagen I (Thermo Fisher Scientific, #A1064401) and added on top of a bottom layer of collagen I. The cells were covered with AIMV medium containing 10% FCS and pre‐activated, primary NK‐cells (250 000 cells per well). After 24 h, spheroids were stained using the Live/Dead™ Viability/Cytotoxicity Kit (ThermoFisher Scientific, #L3224) and imaged using a Zeiss Axiovert S100TV inverted microscope featuring a Fluar 10×/0.5 objective and a Visitron CMOS camera; GFP (green signal; Ex. 470/40, Dichroic mirror T495 LPXR, Em. 525/50) and RFP (red signal; Ex. 545/25, Dichroic mirror T565 LPXR, Em. 605/70) filters were used. Images were acquired using the visiview® software and red signal, staining the dead cells, was analyzed using imagej (NIH, Bethesda, MD, USA [31]).

### 2.5 siRNA‐mediated protein knockdown

Transient knockdown was achieved using small interfering RNA (siRNA). Transfection of three million melanoma cells was conducted using nucleofection (Amaxa Nucleofector, Lonza GmbH, Cologne, Germany) using the SF Cell Line Kit (Lonza, #V4XC‐2024) according to the manufacturer's protocol. The following siRNAs were used for the procedures: Silencer® Select siRNA CIITA (Thermo Fisher, #4392420, AssayID: s8767), CEACAM1 (Thermo Fisher, #4392420, AssayID: s1978), or negative control (Thermo Fisher, #4390843). The cells were used for further analysis after 72 h.

### 2.6 Flow cytometry

MHC II levels were analyzed by staining of 300 000–400 000 melanoma cells with anti‐human APC REAfinity™ HLA‐DR, ‐DP, ‐DQ (REA332 clone, Miltenyi Biotec, 1 : 1000) or with the respective isotype control (APC human recombinant IgG1 isotype control) in staining solution (PBS, 1% FCS, 2 mm EDTA). Staining was performed for 20 min on ice. Samples were acquired on a FACS Canto II (BD Biosciences: San Diego, CA, USA) flow cytometer or a Northern Lights™ Spectral Analyzer (Cytek: Fremont, CA, USA). Quantification of marker expression was conducted by comparison of the mean fluorescence intensities (MFI) using flowjo V10.10.0. Results are presented as netto fluorescence (netMFI), calculated by MFI subtracted by background MFI measured in APC isotype controls.

Primary NK‐cell characterization was performed by blocking the Fc receptor, using Human TruStain FcX™ (BioLegend, #422302). Next, cells were stained with the LIVE/DEAD™ Fixable Aqua Dead Cell Stain Kit (Thermo Fisher, #1091413), according to the manufacturer's protocol. Staining of CD3 (VioBlue, Miltenyi Biotech, #130‐114‐710) and CD56 (PE, Miltenyi Biotech, #130‐113‐312) was performed in staining solution for 20 min on ice in the dark. For intracellular staining of IFNγ (anti‐human IFNγ antibody, APC/Cy7, BioLegend, #1097924), Tue‐Nuclear™ Transcription Factor Buffer Set (Biozol: Hamburg, Germany, #424401) was used according to the manufacturer's protocol.

### 2.7 RT‐qPCR

Total RNA was isolated using NucleoSpin RNA Plus (Macherey‐Nagel: Düren, Germany, #740984.250). To examine gene expression, 800 ng of isolated RNA was reversely transcribed to cDNA using Superscript™ IV Reverse Transcriptase Kit (Thermo Fisher Scientific, #18090010), Ribonuclease Inhibitor (Life Technologies, #EO0382), and Oligo d(T)_12–18_ Primer (Life Technologies, #18418012) according to the manufacturer's protocol. RT‐qPCR was conducted using GoTaq® qPCR Master Mix kit (Promega: Walldorf, Germany, #A26002) and primers (listed below), purchased from Qiagen (Hilden, Germany) or Sigma‐Aldrich, previously used elsewhere [32]. CT values were acquired by Bio‐Rad CFX96 Real‐Time System (Bio‐Rad Laboratories, Feldkirchen, Germany), and CT values of the target mRNAs were normalized to CT values of the housekeeping gene TATA box binding protein (TBP). Analysis of gene expression levels was done using the mxpro software. Data were quantified using the $2^{-\Delta C_{T}}$ method compared to the housekeeping gene TBP.

**Table:** 

Target	Forward primer	Reverse primer
CIITA	5′‐CTGGCTGGAGAAGAAGAGATTG	5′‐AGTTCCGCGATATTGGCATAA
HLA‐DR‐A	5′‐ATCATCCAGGCCGAGTTCTA	5′‐CCGTCTCCTTCTTTGCCATATC
HLA DQ‐A	5′‐CAACACCCTCATTTGTCTTGTG	5′‐CTCAGAAACACCTTCTGTGACT
HLA DPA	5′‐CCCTGAAGACAGAATGTT	5′‐CAAACGCGGCATAAGTTGAC
ULBP2	5′‐CAAGTGCAGGAGCACCACTCG	5′‐CAGATGCCAGGGAGGATGAAGC
ULBP3	5′‐GGAAGAAGAGGCTGGAACCC	5′‐TCAGATGCCAGGGAGGATGA
IRF1	5′‐CTCTGAAGCTACAACAGATGAGG	5′‐CTGTAGACTCAGCCCAATATCCC
IRF2	5′‐GAGTATGCGGTCCTGACTTCAAC	5′‐CATCGCTGGGCACACTATCAGTCG
USF1	5′‐TCGTGCAGCTCTCCAAGATAATCC	5′‐CCTGTTGTCGAAGCACGTCATTG
CEACAM1	5′‐TCTACCCTGAACTTTGAAGCCCA	5′‐TGAGAGACTTGAAATACATCAGCACTG
LGALS9	5′‐GTCTCCAGGACGGACTTCAG	5′‐CGTACCCTCCATCTGGTTCC
TBP	5′‐CGGAGAGTTCTGGGATTGT	5′‐GGTTCGTGGCTCTCTTATC

### 2.8 ELISA

Secreted cytokines (IFNγ) were detected in NK‐cell/melanoma co‐cultures using the Human IFN gamma Uncoated ELISA kit (Invitrogen, #88‐7316) according to the manufacturer's protocol in 96‐well plates. After the procedure, absorbance was read at 450 and 570 nm for wavelength subtraction in a microplate reader (CLARIOstar®, BMG Labtech).

### 2.9 Single‐cell RNA‐sequencing analysis of melanoma samples

Single‐cell RNA‐sequencing data from human acral melanoma, cutaneous melanoma, and a single lymph node metastasis were obtained from GSE215120 [33] excluding one post‐treatment sample. Additional samples, including melanoma brain metastasis and cutaneous melanoma, were provided by GSE174401 [34]. Cerebrospinal fluid samples were excluded from the analysis, and only patients not undergoing immune therapy at the time point of sample collection were considered.

Cells were filtered by mitochondria content (≤ 5%), UMI counts (> 1000) and number of expressed genes (> 400 & < 6000). Doublets were removed using doubletfinder v2.0.4 [35]. Expression values were normalized using log normalization, as implemented in seurat v5.1.0 [36]. Subsequently, dimensionality reduction was performed using the UMAP algorithm, followed by further visualization steps.

After merging the datasets, cell types were annotated using scannotatr v1.10.0 [37] for all available cell types and filtered for immune cells and melanocytes. Cells were identified as melanoma cells based on MLANA expression or classified as melanocytes. NK‐cells were characterized by the application of the following marker genes: IL‐32, TBX21, PTPRC, NKG7, KLRB1, CD27, XCL2, NCAM1, CD8a, CD3d, CD8b, CD7, NCR1, and CD3e. The predicting probability threshold was set to 0.5, and no parent model was applied. Lastly, cellchat v2.1.2 [38] with standard parameters was used to interfere cell–cell communication pathways.

In brief, cellchat creates intercellular communication networks using curated databases of ligand–receptor pairs and cofactors. The strength of interaction between two cell types is determined by the average expression of ligands in one cell type and receptors in the other, evaluated using the Hill function and simplified principles from mass action models. The statistical significance of these interactions is assessed through random permutation testing.

### 2.10 Statistical analysis

Statistical analyses were performed using prism 9 software (GraphPad: Boston, MA, USA). A *P* value of < 0.05 was considered significant. Statistical tests and sample sizes (*n*) are stated in figure legends.

## 3 Results

### 3.1 Melanoma cells co‐cultured with primary NK‐cells develop resistance to NKmK

To assess the effect of NK‐cells on melanoma resistance, we used an *in vitro* co‐culture system of melanoma cells with primary NK‐cells. NK‐cells from healthy donors were isolated and activated with interleukin‐2 (IL‐2). To ensure enrichment of NK‐cells from peripheral blood mononuclear cells (PBMCs), we performed classical NK‐cell characterization using CD3^−^ and CD56^+^ expression patterns, analyzed by flow cytometry, demonstrating a pure NK‐cell culture (97%, Fig. S1A–H). To assess the effects of NK‐cell interactions on melanoma cells, we selected four genetically diverse melanoma cell lines that originate from different disease stages. Specifically, WM1366 and WM793 were established from primary melanoma lesions in the vertical growth phase, while 1205Lu and WM3734 were derived from distant metastatic sites (lung and brain, respectively). Notably, 1205Lu originates from WM793, providing a well‐matched system comparing primary and metastatic melanoma within a simplified setting. Furthermore, the selected cell lines display distinct mutational profiles, including differences in BRAF and NRAS status (BRAF V600E: 1205Lu, WM793, WM3734 and NRAS Q61L: WM1366), further supporting their use for studying the impact of NK‐cell interactions across genetically diverse melanoma contexts.

**Fig. 1:**
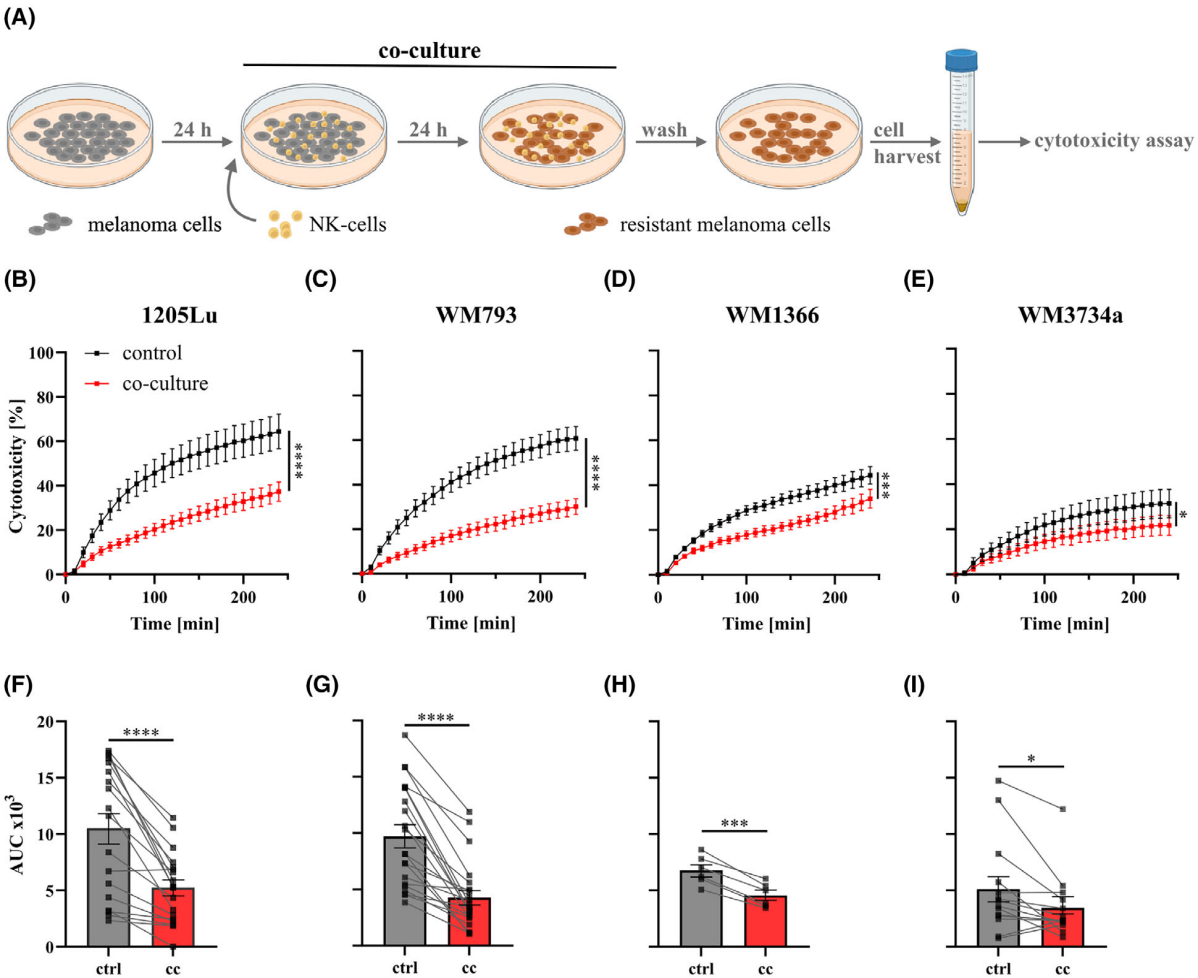
Resistant phenotype of melanoma cells to NKmK after co‐culture. (A) Schematic of co‐culture‐based induction of resistant melanoma cells. Created with BioRender.com. (B–E) Real‐time cytotoxicity assay of different melanoma cell lines with primary NK‐cells, demonstrating a resistant phenotype of previously co‐cultured melanoma cells. (B) 1205Lu, *n* = 18; (C) WM793, *n* = 21; (D) WM1366, *n* = 6; (E) WM3734a, *n* = 14, data presented as mean ± SEM. (F–I) Area under the curve (AUC) after 4 h of primary NK‐cells targeting control (ctrl) or co‐cultured (cc) melanoma cells, each dot represents a single NK‐cell donor. (*) *P* < 0.05; (**) *P* < 0.01; (***) *P* < 0.001; (****) *P* < 0.0001; assessed by two‐tailed paired Student's *t*‐test.

For co‐culture, melanoma cells were seeded and NK‐cells were added at a 1 : 1 ratio for 24 h. Surviving melanoma cells were used for phenotype characterization as depicted in Fig. 1A. Using a real‐time killing assay [25, 29], we analyzed the susceptibility of genetically diverse melanoma cells to NK‐cell‐mediated killing (NKmK) over 4 h (Fig. 1B–I). We previously demonstrated that genetically distinct melanoma cells exhibit varying susceptibility to NKmK [25]. In this study, we observed that melanoma cells co‐cultured with NK‐cells for 24 h exhibited decreased susceptibility to NKmK. This NK‐cell‐induced resistance was evident in melanoma cells originating from primary lesions (WM1366, WM793) and from metastatic melanoma sites (1205Lu, WM3734). Although the killing efficiency varied between NK‐cell donors (Fig. 1F–I), our results consistently demonstrated that co‐culture caused a decrease in NKmK, which was independent on the initial susceptibility.

To evaluate the persistence of this induced resistance, NK‐cells were removed after 24 h of co‐culture and melanoma cells were allowed to recover for up to 72 h. NKmK was assessed at 24‐h intervals (Fig. S1I). As shown in Fig. S1J,K, previously co‐cultured and NKmK‐resistant melanoma cells (1205Lu and WM793) exhibited a gradual decline in resistance, progressively regaining susceptibility to NKmK over time. Therefore, the observed co‐culture‐induced suppression of NKmK was reversible, highlighting a clear distinction from resistance mechanisms typically associated with targeted therapies [39]. For simplicity, we refer to the induced decrease of the susceptibility to NKmK as ‘resistance’ throughout the manuscript.

### 3.2 Bioinformatic analyses reveal genes and pathways involved in NKmK resistance

To analyze potential molecular mechanisms of melanoma resistance formation, we sought to study prominent interaction pathways between melanoma cells and immune cells from patient data sets. We analyzed single‐cell RNA‐sequencing data of 15 melanoma samples [33, 34] from patients suffering from cutaneous and acral melanoma with brain and lymph node metastasis. Only patients who were not on treatment were considered for further evaluation. To emulate the co‐culture setup, immune and melanoma cells of all patients were combined and analyzed together after applying pre‐processing and quality control steps.

**Fig. 2:**
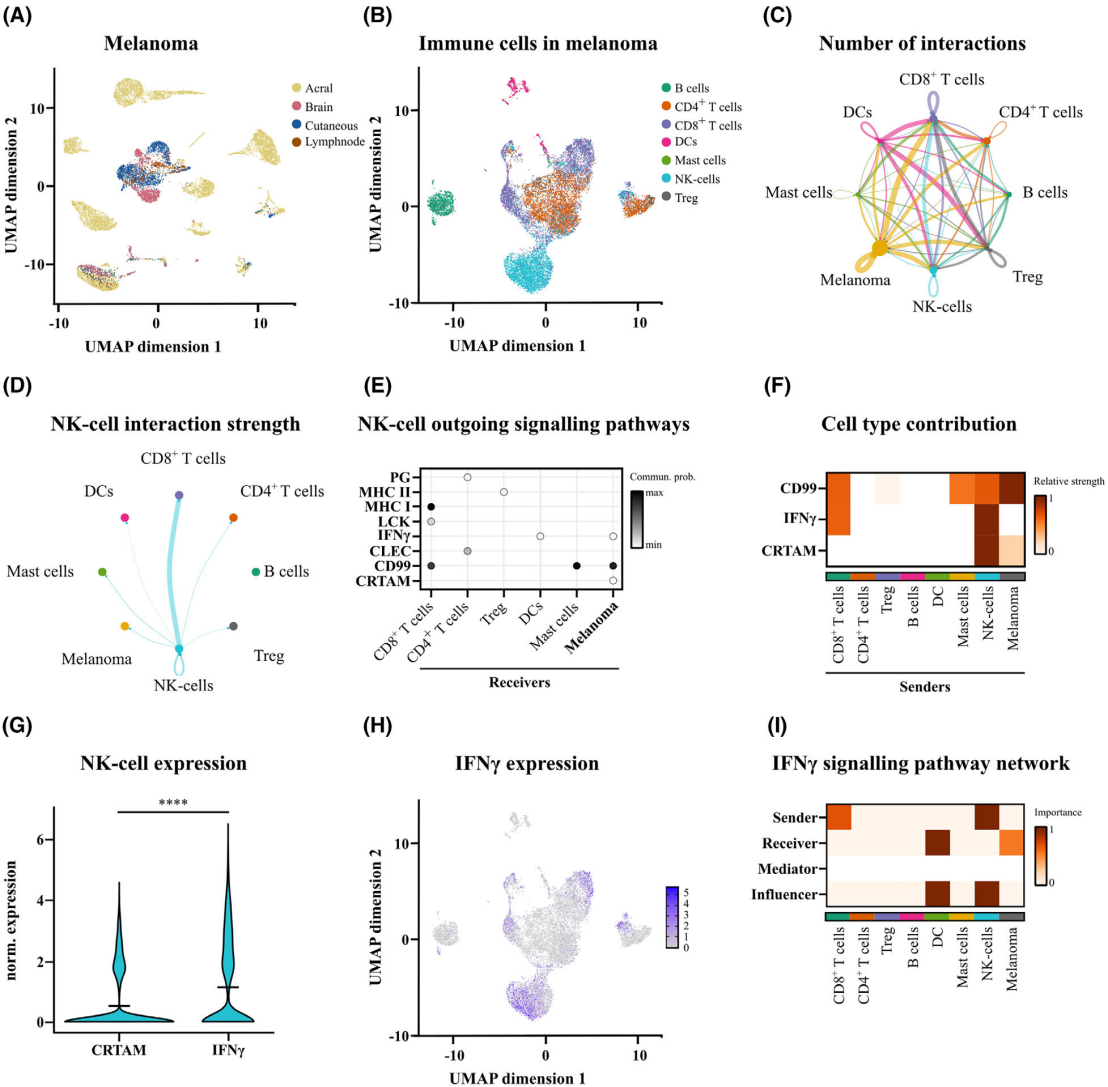
NK‐cell–melanoma communication pathways derived from scRNA‐seq data by integrating melanoma patient samples. UMAP plots illustrating the (A) melanoma and (B) immune cell landscape within melanoma tissue. (C) Signaling network depicting interactions between immune and melanoma cells. Arrows thickness represents the number of signaling pathways. (D) Signaling strength of outgoing signaling pathways from NK‐cells. Arrows thickness represent the estimated signaling strength. (E) Outgoing NK‐cell signaling pathways show three pathways that potentially influence melanoma‐NK‐co‐cultures. (F) Comparison of the relative strength of NK‐cell–melanoma communication pathways across immune cell types. (G) Expression of the CRTAM and IFNγ pathway relevant genes in NK‐cells, mean indicated by the dash (log_2_ fold change IFNγ/CRTAM: 0.61). (****) *P* < 0.0001; assessed by two‐tailed Mann–Whitney *U*‐test. (H) IFNγ gene expression in the immune cell landscape within the melanoma tissue. Scale indicates the normalized expression values. (I) IFNγ signaling within melanoma tissue indicate NK‐cells as major IFNγ source. Data obtained from [33, 34].

The standardized data were further reduced using the UMAP algorithm, divided by melanoma (Fig. 2A) and the immune compartment (Fig. 2B) and were annotated based on the cell type. As depicted, we identified a range of immune cells, including B cells, T cells, mast cells, dendritic cells (DCs), and NK‐cells. Interestingly, immune cells formed distinct clusters, while melanoma cells from different origins exhibited high heterogeneity across different clusters (Fig. 2A,B). Next, a communication network of well‐known ligand–receptor pairs between the cell types was estimated by using cellchat2 and suggested a highly interactive system (Fig. 2C). To this end, melanoma cells and NK‐cells displayed direct interactions with outgoing signals from both cell types. Of note, similar interactions of NK‐cells were observed also with other cell types (Fig. 2D). As we aimed to identify key molecular players involved in melanoma resistance to NK‐cell cytotoxicity, we focused on the outgoing signals from NK‐cells that could directly influence melanoma cells. All signaling pathways predicted using cellchat2 were analyzed and visualized [38], revealing multiple factors regulated by NK‐cells across different cell types (Fig. 2E). Focusing on the NK‐cell–melanoma interaction, we identified three primary regulatory mechanisms: IFNγ, a cytokine secreted by NK‐cells that plays a crucial role in modulating melanoma cell responses [5], as well as CD99 and CRTAM, two surface proteins involved in cell adhesion [40, 41, 42] (Fig. 2E). We further analyzed these pathways within the entire interactome, assessing the relative strength of outgoing signals from different tumor components to determine their relevance within the system. This analysis identified IFNγ and CRTAM as the strongest signals originating from NK‐cells (Fig. 2F). IFNγ signals were exclusively derived from NK‐cells and CD8^+^ T lymphocytes, whereas CRTAM signals were linked to both NK‐cells and melanoma cells. Since CD99 signaling can be initiated by other tumor components, in addition to melanoma cells (predicted to generate the strongest signals), we focused our analysis on CRTAM and IFNγ expression in NK‐cells. Our findings revealed that IFNγ gene expression was significantly higher than CRTAM in NK‐cells (Fig. 2G). Reviewing the gene expression of IFNγ within the immune compartment confirms high expression patterns in NK‐cells (Fig. 2H), as predicted previously (Fig. 2F). Consequently, we further examined IFNγ signaling, not only in terms of outgoing signals but also by identifying the key receiving components (Fig. 2I). As previously shown in Fig. 2F,H, NK‐cells and CTLs were the primary sources of IFNγ, with NK‐cell‐derived signaling appearing more pronounced. Additionally, NK‐cells and dendritic cells (DCs) exerted the strongest influence on IFNγ signaling. Apart from DCs, melanoma cells were predicted to be the only cells receiving IFNγ signals (Fig. 2I).

Summarized, these data demonstrate the importance of melanoma and NK‐cell interaction, with IFNγ signaling being one of the main signaling pathways mediating the interaction between melanoma and NK‐cells. We concluded that IFNγ signaling and, therefore, its secretion plays a pivotal role in melanoma resistance formation during melanoma and NK‐cell interaction, that is, the melanoma tumor microenvironment.

### 3.3 Melanoma cells are exposed to IFNγ during NK‐cell co‐culture

**Fig. 3:**
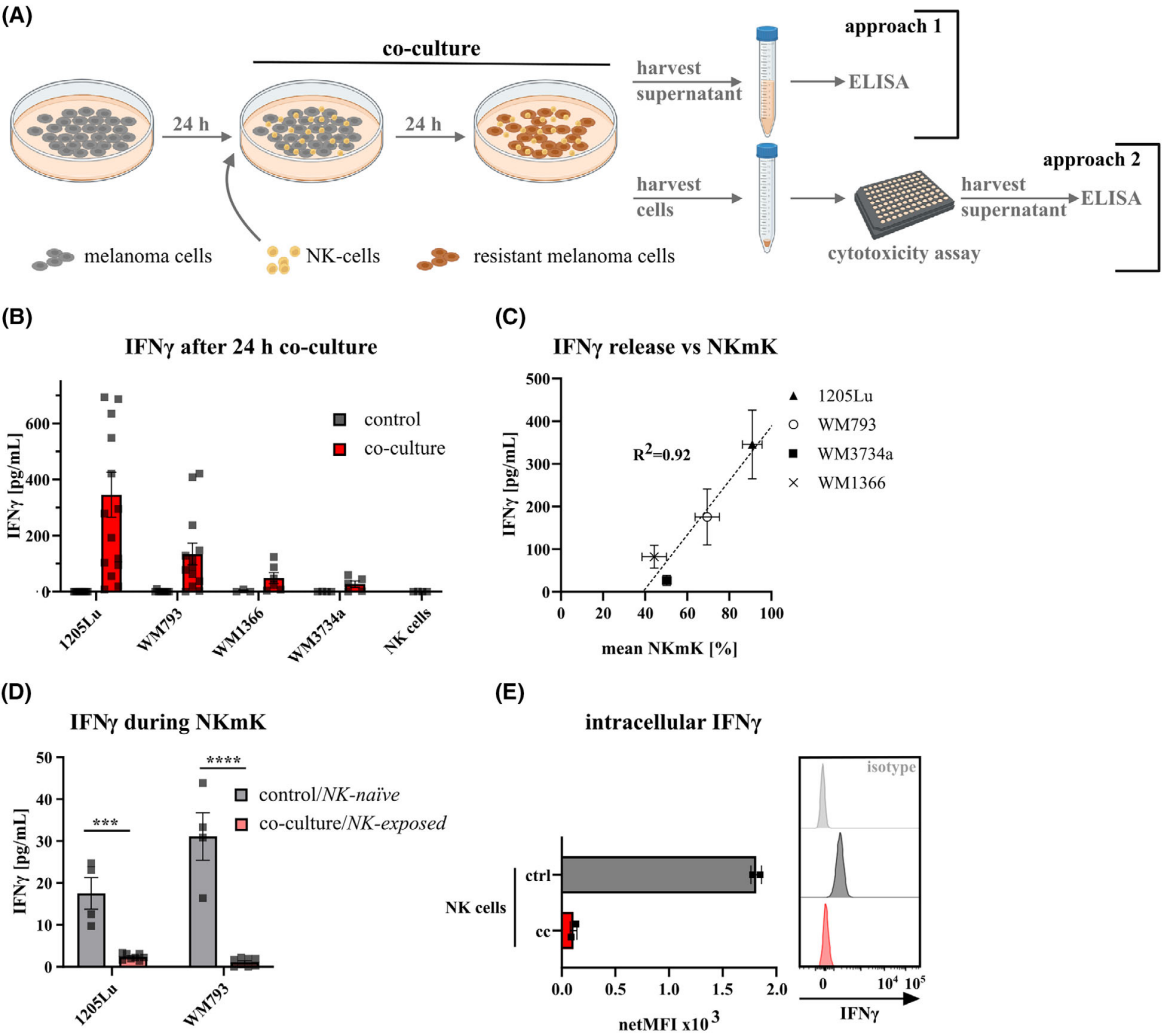
Melanoma cells are exposed to IFNγ during NK‐cell co‐culture. (A) Schematic of IFNγ measurements after co‐culture, IFNγ content was measured after 24 h co‐culture (approach 1) and after performing the cytotoxicity assay post‐co‐culture (approach 2). Image created with BioRender.com. (B) ELISA detecting IFNγ in the media of control melanoma cells (*NK‐naïve*, gray) or after 24 h co‐culture (*NK‐exposed*, red, approach 1); 1205LU (*n* = 15), WM793 (*n* = 13), WM1366 (*n* = 6), and WM3734a (*n* = 5). (C) NKmK of control melanoma cells correlated to IFNγ amount after 24 h co‐culture, *R* ^2^ = 0.92. (D) IFNγ content after performing a 4 h cytotoxicity assay on control/*NK‐naïve* (gray, *n* = 4) or co‐cultured/*NK‐exposed* cells (red, *n* = 8) using two NK‐cell donors (approach 2). (E) Flow cytometric analysis of intracellular IFNγ in NK‐cells from single culture (ctrl) or after 4 h co‐culture with WM793 melanoma cells (cc). (***) *P* < 0.001; (****) *P* < 0.0001; assessed by two‐tailed paired Student's *t*‐test.

In order to decipher the role of IFNγ in our co‐culture‐based model system, we measured IFNγ (using Enzyme‐linked Immunosorbent Assay (ELISA)) secretion after 24‐h co‐culture of melanoma cells and NK‐cells (Fig. 3A; approach 1) and performed cytotoxicity assays of *NK‐naïve* (control) melanoma cells and *NK‐exposed* (co‐cultured) melanoma cells using fresh primary NK‐cells (Fig. 3A; approach 2). After 24 h co‐culture we determined the secreted IFNγ and found that during co‐culture, all four melanoma cell lines were exposed to IFNγ (Fig. 3B). However, the amount of secreted IFNγ varied per melanoma cell line, whereby IFNγ levels during co‐culture correlated with the initial NKmK susceptibility of the four melanoma cell types (Fig. 3C). Given this important finding, we next determined IFNγ secretion in co‐culture conditions (4 h) in which we exposed *NK‐naïve* (control) and *NK‐exposed* (co‐cultured) 1205Lu and WM793 melanoma cells to fresh primary NK‐cells. As demonstrated in Fig. 3D, we observed a strong reduction of IFNγ production in the co‐culture containing *NK‐exposed*, that is, resistant melanoma cells as compared to the co‐cultures containing *NK‐naïve* melanoma cells. Flow cytometric analysis of primary NK‐cells from a single culture demonstrated the presence of intracellular IFNγ (Fig. 3E), whereas no IFNγ was detected in the supernatant (Fig. 3A). However, post‐co‐culture, NK‐cells lacked detectable intracellular IFNγ (Fig. 3E), thus supporting the well‐documented knowledge that NK‐cells are the principal source of IFNγ secretion within these co‐culture systems [43, 44, 45].

In summary, these findings indicate a novel and highly significant regulatory constellation within NK‐cell‐based immune responses against melanoma. They uncover a co‐regulatory feedback mechanism in which the initial sensitivity of a specific melanoma cell subtype plays a decisive role in determining the final outcome; namely, the potential susceptibility of each individual tumor or cancer subtype to NK‐cell‐mediated cytotoxicity.

### 3.4 IFNγ is the key player of melanoma resistance formation to NKmK

**Fig. 4:**
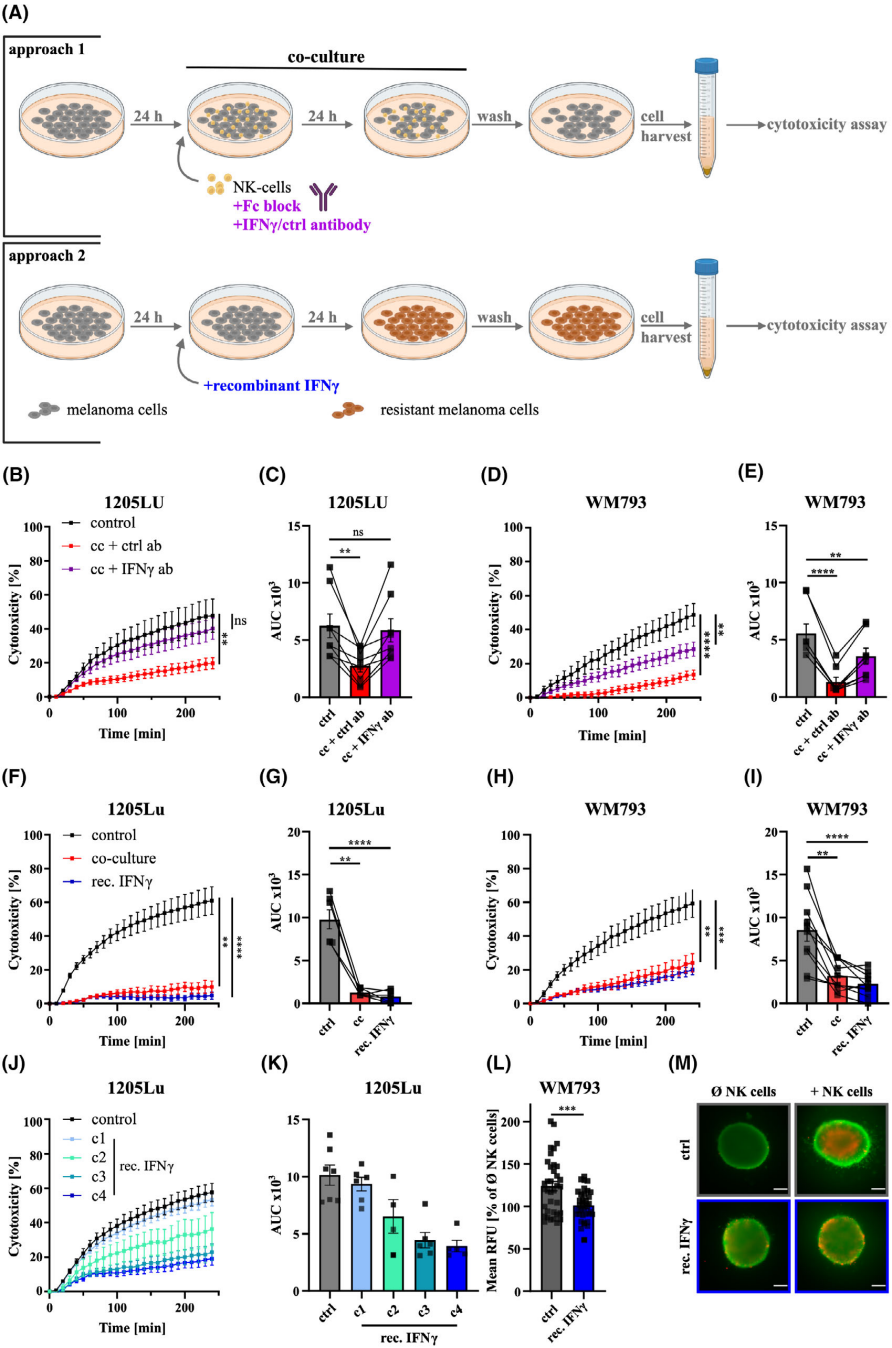
IFNγ regulates NK‐cell‐induced melanoma resistance formation to NKmK. (A) Schematic of co‐culture‐based model system with Fc block and anti‐IFNγ‐blocking antibody during co‐culture (approach 1) or recombinant IFNγ instead of co‐culture (approach 2), image created with BioRender.com. (B–E) Cytotoxicity assays of control (ctrl) or co‐cultured (cc) melanoma cells, treated with isotype or IFNγ‐blocking antibody (ctrl, *n* = 6; cc + ctrl ab/IFNγ ab, *n* = 8). (B) 1205Lu killing kinetics with (C) quantified AUC, each dot represents a biological replicate. (D) WM793 killing kinetics with (E) quantified AUC. (F–I) Cytotoxicity assays of control (ctrl), co‐cultured (cc), or IFNγ‐treated (rec. IFNγ) cells, (F) 1205Lu killing kinetics (ctrl, rec. IFNγ *n* = 6; cc *n* = 2) with (G) quantified AUC. (H) WM793 killing kinetics (ctrl, rec. IFNγ *n* = 10; cc *n* = 6) with (I) quantified AUC. Cytotoxicity assays of 1205Lu control (ctrl) or IFNγ‐treated cells with the concentrations c1 (35 pg·mL^−1^), c2 (350 pg·mL^−1^), c3 (3.5 ng·mL^−1^), and c4 (350 ng·mL^−1^) with (J) killing kinetics and (K) quantified AUC. (L, M) *3D* NKmK of WM793 spheroids with primary NK‐cells. (L) Quantification of dead signal in *3D* NKmK of WM793 normalized to spheroid controls without NK‐cells; WM793 rec. IFNγ spheroids were treated for 24 h with IFNγ and compared to control (ctrl) cells. Each dot represents a single spheroid, co‐cultures were performed with four different donors. (M) Representative images of *3D* cytotoxicity assay of WM793; red: dead cells; green: live cells; scale bar 200 μm; gray frame (top): control WM793 spheroids; blue frame (bottom): rec. IFNγ‐treated WM793 spheroids; left: without NK‐cells; right: with NK‐cells. Data presented as mean ± SEM. Statistical analysis by two‐tailed paired Student's *t*‐test of the AUC compared to control cells; (ns) *P* > 0.05; (**) *P* < 0.01; (***) *P* < 0.001; (****) *P* < 0.0001.

Next, we focused on understanding the molecular details of IFNγ‐mediated melanoma cell resistance formation. For this, we applied monoclonal antibodies to block IFNγ activity during NK‐cell–melanoma cell co‐culture (see Fig. 4A; approach 1) or by adding recombinant IFNγ to melanoma cells instead of using primary NK‐cells as a source (Fig. 4A; approach 2).

Within the first approach, we initially performed an Fc block to inhibit the interaction of CD16 with the Fc part of the monoclonal antibodies. Next, NK‐cells were added to the melanoma cell culture, with either the IFNγ‐blocking antibody or with an isotype control. Following 24 h of co‐culture, the control‐treated 1205Lu and WM793 cells displayed a resistant phenotype, as expected. However, the presence of the IFNγ‐blocking antibody caused a significant recovery of both the 1205Lu and WM792 melanoma cell susceptibility to NKmK (Fig. 4B–E). Furthermore, in order to mimic NK‐cell co‐culture using recombinant IFNγ, we performed experiments as depicted in the second approach of Fig. 4A. To this end, we treated 1205Lu and WM793 melanoma cells with either recombinant IFNγ (350 ng·mL^−1^) or with primary NK‐cells in a co‐culture for 24 h. Again, both 1205Lu as well as WM793 developed resistance to NKmK after co‐culture with NK‐cells. Notably, a very similar behavior, that is, resistance to NKmK, could be observed when the NK‐cell co‐culture was replaced by IFNγ (Fig. 4F–I). We further identified a concentration‐dependent effect of IFNγ on NKmK, with lower IFNγ concentrations inducing reduced resistance formation in 1205Lu melanoma cells (Fig. 4J,K). To examine the role of IFNγ in melanoma susceptibility to NKmK within a more physiologically relevant context, we adapted the *2D in vitro* cytotoxicity assay to a *3D* spheroid model. Concretely, we evaluated the viability of melanoma spheroids (embedded in a collagen matrix) exposed to NK‐cells, either as untreated controls or following IFNγ treatment. Obviously, pretreatment of WM793 spheroids with IFNγ significantly decreased NK‐cell‐induced cell death upon (Fig. 4L,M), thus corroborating our findings from the *2D* assays.

These findings provided novel insights regarding the regulation of NKmK and supported previous observations regarding the central role of IFNγ in this context.

### 3.5 IFNγ controls MHC II and CIITA expression

**Fig. 5:**
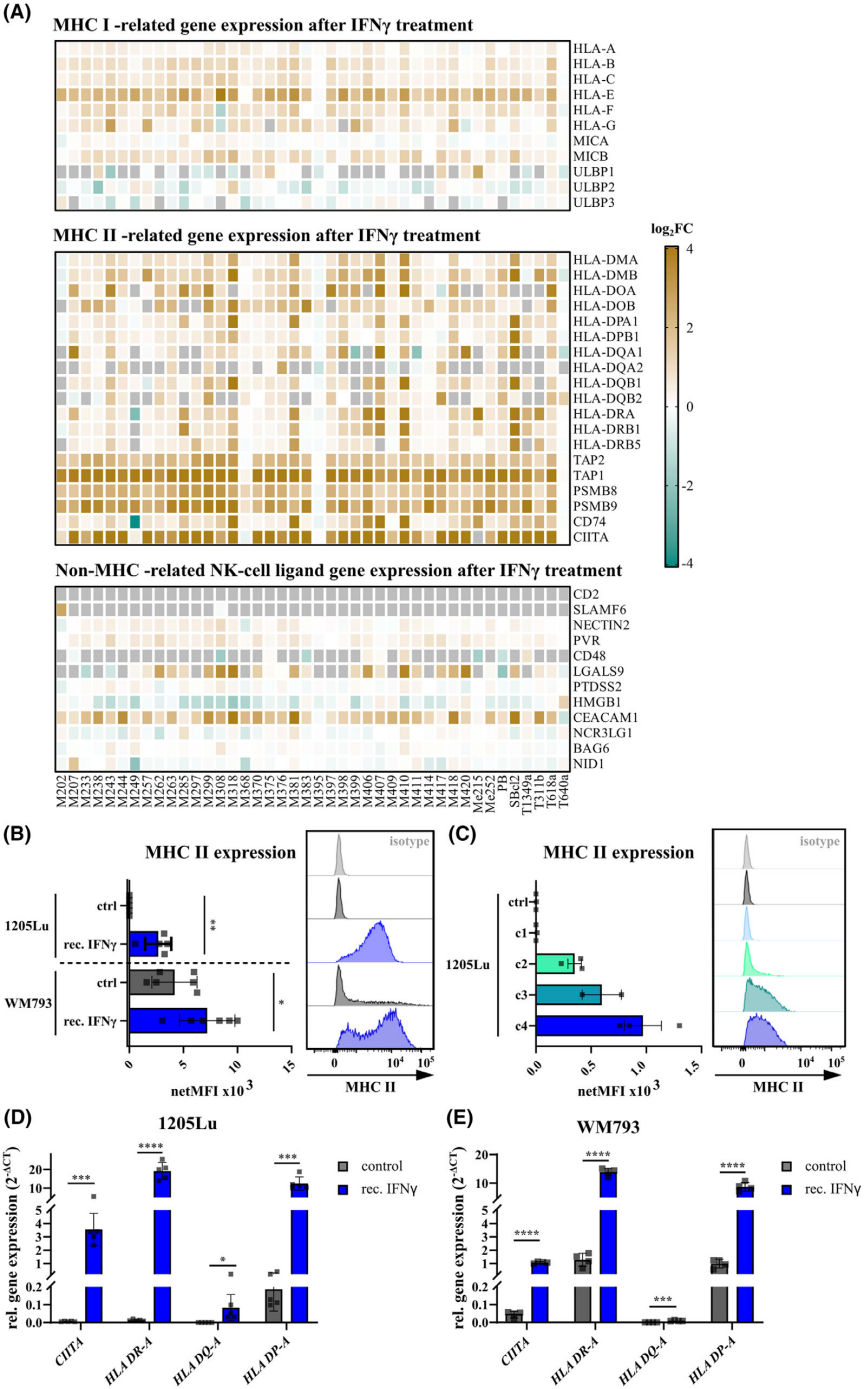
IFNγ regulates MHC II‐related genes in melanoma. (A) Impact of IFNγ on melanoma gene expression. Heatmaps displaying log2 fold changes of melanoma cell genes after IFNγ treatment [46], focusing on NK‐cell ligands. Highest panel: MHC I‐related genes, middle panel: MHC II‐related genes, lowest panel: non‐MHC‐related genes. (B) Flow cytometric analysis of MHC II isotypes (HLA‐DR, ‐DQ, ‐DP) of (B) control (ctrl) melanoma cells or after treatment with IFNγ (rec. IFNγ) (1205Lu, *n* = 6; WM793, *n* = 5) and of (C) 1205Lu, treated with different concentrations of rec. IFNγ: c1 (35 pg·mL^−1^, *n* = 3), c2 (350 pg·mL^−1^, *n* = 3), c3 (3.5 ng·mL^−1^, *n* = 2), and c4 (350 ng·mL^−1^, *n* = 3). Histograms show representative MHC II expression of the corresponding samples. (D, E) Gene expression analysis of MHC II isotypes *HLA‐DR*, *‐DQ*, *‐DP* and *CIITA* of (D) 1205Lu (*n* = 5) and (E) WM793 (*n* = 4); each dot represents a biological replicate. Statistical analysis by two‐tailed unpaired Student's *t*‐test compared to control cells, (*) *P* < 0.05; (**) *P* < 0.01; (***) *P* < 0.001; (****) *P* < 0.0001.

Our findings confirmed and further expanded the understanding of IFNγ's role in melanoma cell resistance to NKmK. However, the precise molecular mechanisms and targets involved in this process remained not fully understood. To address this, we extended our study by investigating the impact of IFNγ on melanoma gene expression and identifying potential molecular targets contributing to IFNγ‐induced resistance. For this purpose, we utilized a publicly available RNA‐seq dataset comprising 42 melanoma cell lines before and after IFNγ exposure [46]. To identify genes that might be involved in controlling the sensitivity of melanoma cells to NK‐cell‐mediated cytotoxicity, we used a list of NK‐cell ligand genes that can regulate NK‐cell activity [47], including MHC I and MHC II genes. As depicted in Fig. 5A, a number of NK‐cell relevant genes were regulated by IFNγ in most of the examined melanoma cell lines. This analysis identified MHC I‐related genes (Fig. 5A, highest panel), thus corroborating observations from several previous studies [17, 48]. However, we also identified numerous MHC II‐related genes, including their transcriptional master regulator, class II transactivator (CIITA), to be tightly controlled by IFNγ. Specifically, most of these genes were markedly upregulated following IFNγ exposure in nearly all melanoma cell lines (Fig. 5A, middle panel). Additionally, we observed several MHC‐independent NK‐cell ligands to be also regulated by IFNγ (Fig. 5A, lower panel).

Referring to the patient datasets presented in Fig. 2, we performed additional differential gene expression analysis among the patient melanoma cell samples (Fig. S2A–C). To this end, we considered the contribution of the IFNγ receptors 1 and 2 (IRNGR1/2) [49]. Accordingly, we evaluated the expression patterns of IFNGR1/2 within the melanoma landscape and observed significant expression in almost all clusters (Fig. S2A). Moreover, this analysis indicated that MHC I was expressed ubiquitously between the cell clusters (Fig. S2B). Notably, the MHC II expression was also evident but was more heterogeneous between the melanoma cell clusters (Fig. S2C).

In contrast to MHC I, MHC II is currently not recognized as a major regulator of NK‐cell‐mediated immune responses. Also, the present knowledge about the MHC II regulation by IFNγ is scarce. Accordingly, we next focused on elucidating the contribution of the IFNγ‐CIITA‐MHC II axis on melanoma cell resistance to NKmK. To achieve that, we exposed 1205Lu and WM793 melanoma cells to recombinant IFNγ and tested for their protein and gene expression patterns. Both cell lines exhibited a marked increase in MHC II surface protein expression following IFNγ treatment, thereby confirming the findings derived from the Grasso et al. dataset (Fig. 5B). Similar to our findings regarding the melanoma susceptibility to NKmK upon IFNγ exposure (Fig. 4J,K), we find the upregulation of MHC II surface expression to be dependent on the initial IFNγ concentration in 1205Lu (Fig. 5C). In addition, RT‐qPCR analyses of IFNγ‐exposed melanoma cells revealed upregulated expression of *CIITA* and several MHC II‐related genes (*DR‐A*, *DQ‐A*, *DP‐A*, Fig. 5D,E).

Furthermore, we aimed to validate other IFNγ‐regulated melanoma cell ligands, apart from MHC II‐related genes. As depicted in Fig. 5A, the UL16 binding protein 2 and 3 (*Ulbp2/3*), carcinoembryonic antigen‐related cell adhesion molecule 1 (*Ceacam1*) and galectin 9 (*LgalS9*) were identified as possible regulators of melanoma cell resistance to NK‐cell cytotoxicity (Fig. S2A–D). ULBP2 and ULBP3 belong to the group of unconventional MHC I molecules and have been reported to activate NK‐cell activity through interactions with NKG2D receptors [50]. NKG2D receptors can bind a variety of ligands but are generally known to be one of the main triggers for NK‐cell activity [51]. Therefore, we expected that IFNγ exposure would lead to a downregulation of these ULBP genes, resulting in decreased NKmK. While *Ulbp2* was downregulated after IFNγ exposure in 1205Lu, this effect was not observed in WM793 (Fig. S2A). For *Ulbp3*, on the other hand, no changes between the conditions were observed in both melanoma cell lines (Fig. S2B). Additionally, analysis of patient data sets only displayed minor expression of *ULBP2/3* among all melanoma cell clusters (data not shown). CEACAM1 is a transmembrane glycoprotein, which can be expressed on melanoma and immune cells [52]. Evasion of NK‐cell‐mediated cytotoxicity in melanoma cells has been proposed to occur through the homophilic binding of CEACAM1 on melanoma cells and NK‐cells [53]. Indeed, we found *Ceacam1* gene expression to be upregulated in 1205Lu and WM793 after IFNγ treatment (Fig. S2C), a finding that suggested a possible existence of an alternative mechanism of melanoma resistance formation to NK‐cells. Lastly, we tested for *Lgals9* expression, which was also shown to be upregulated after IFNγ treatment, even though expression levels were generally low (Fig. S2D). Since our findings suggested a possible involvement of CEACAM1 in melanoma resistance formation, we examined its role in the IFNγ‐induced melanoma cell transformation. We used siRNA to target *Ceacam1* and achieved a high knockdown efficiency in 1205Lu and WM793 untreated and IFNγ treated cells (Fig. S2E). To evaluate the functional relevance, we performed NK‐cell‐mediated cytotoxicity assays with control (Ctrl) and CEACAM1 knockdown (si) cells, which were either not treated (wo) or treated with IFNγ (IFNγ). Downregulation of CEACAM1 was expected to, at least to a certain extent, rescue the IFNγ‐induced resistance. However, our results did not indicate a significant increase in NKmK in CEACAM1 knockdown melanoma cells (Fig. S2F). It should be noted here that we were not able to validate the downregulation of CEACAM1 surface expression, which suggested that the protein levels were not affected despite the efficient reduction of *Ceacam1* transcripts. Furthermore, CEACAM1 on melanoma cells was previously demonstrated to interact with CEACAM1 on NK‐cells, which in turn upregulates CEACAM1 in the diseased patient [53]. Given that for this study we used exclusively primary NK‐cells from healthy donors, we cannot exclude the role of CEACAM1 in NKmK in melanoma patients despite the negative outcome of our experiments.

In summary, we identified and validated genes that are controlled by IFNγ in melanoma cells and are involved in regulating their sensitivity to NK‐cell‐mediated cytotoxicity.

### 3.6 Co‐culture with NK‐cells alters gene expression and protein abundance in melanoma cells

**Fig. 6:**
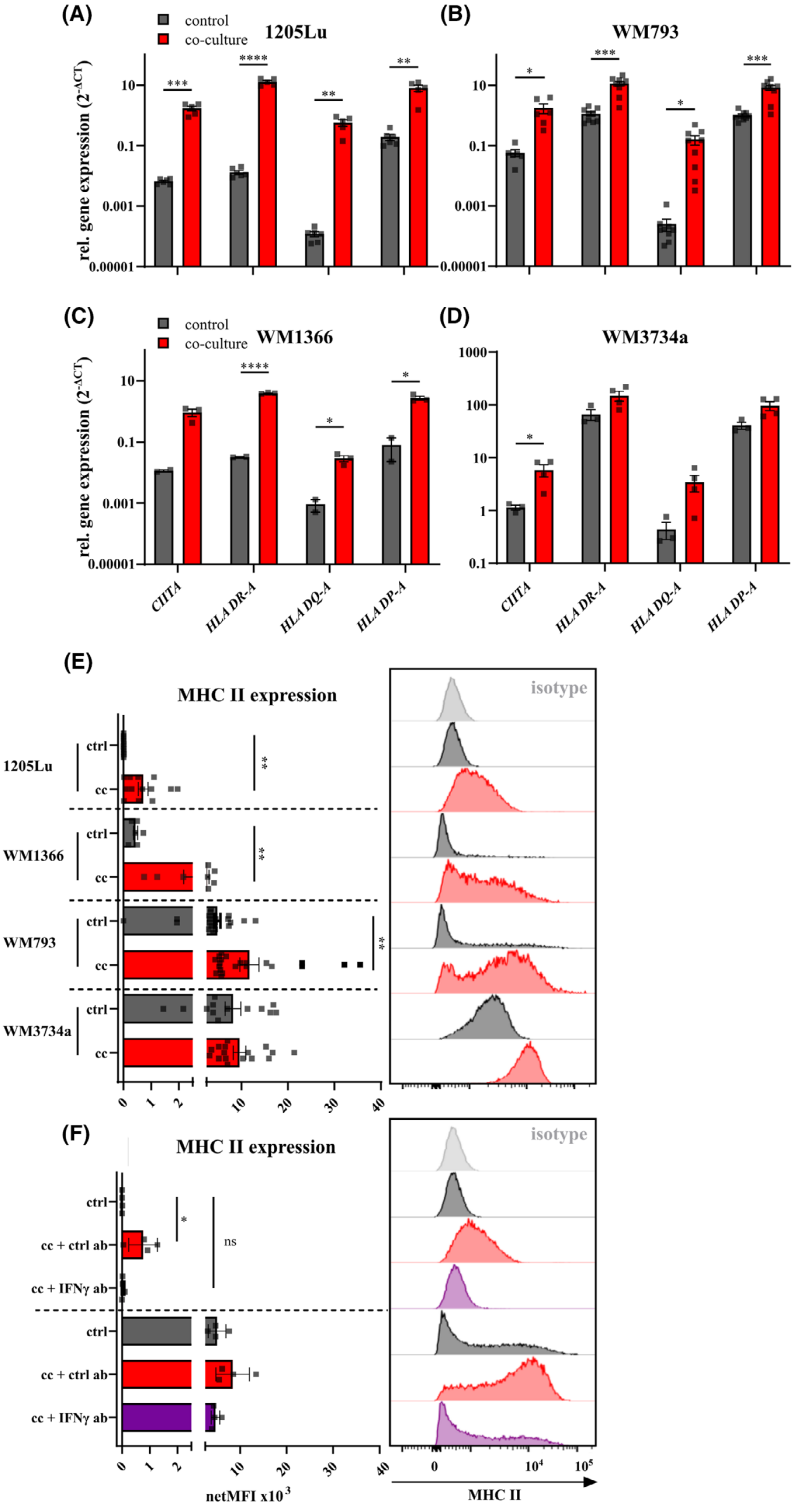
NK‐cell co‐culture alters MHC II expression of melanoma cells. Gene expression analysis of MHC II‐related genes and *CIITA* by RT‐qPCR in (A) 1205Lu (*n* = 5 donors), (B) WM793 (*n* = 9 donors), (C) WM1366 (*n* = 3 donors), and (D) WM3734a (*n* = 4 donors). Expression of *CIITA*, *HLA DR‐A*, *DQ‐A*, and *DP‐A* is increased after 24 h co‐culture with primary NK‐cells (red) compared to control (gray) cells. (E) Increased MHC II expression of co‐cultured (cc) cells compared to control (ctrl). 1205Lu (*n* = 11), WM1366 (*n* = 8), WM793 (*n* = 20), WM3734a (*n* = 6). (F) Flow cytometric analysis of ctrl or cc cells, treated with an IFNγ‐blocking antibody or respective isotype control (*n* = 4). Histograms show representative MHC II expression of the corresponding melanoma cell line. (ns) *P* > 0.05; (*) *P* < 0.05; (**) *P* < 0.01; (***) *P* < 0.001; (****) *P* < 0.0001; assessed by two‐tailed unpaired Student's *t*‐test.

To further examine the link between the influence of recombinant IFNγ treatment and NK‐cell co‐culture in melanoma cells, we determined MHC II protein and gene expression patterns. A RT‐qPCR‐based evaluation of different MHC II molecules indicated that all analyzed MHC II isotypes (*DR‐A*, *DQ‐A*, *DP‐A*) are upregulated after co‐culture with NK‐cells in all melanoma cell lines analyzed (Fig. 6A–D). Similar as MHC II genes, *CIITA* was also significantly higher after co‐culture in the analyzed melanoma cells (Fig. 6A–D). On a protein level, we confirmed the qPCR data and found that in the NK‐cell co‐cultured cells the MHC II surface expression is elevated as compared to the control cells. Notably, the endogenous, unstimulated MHC II expression (comparing the control melanoma cells of 1205Lu, WM1366, WM793, and WM3734 in Fig. 6E) was variable in the melanoma cells used for this study. Nevertheless, irrespective of the basal expression levels, an increase in MHC II expression following co‐culture was observed across all cell lines (Fig. 6E).

To examine the role of endogenous CIITA activity on MHC II expression, we performed knockdown (KD) experiments using siRNA targeting *CIITA* in 1205Lu (low basal MHC II expression) and WM793 (high basal MHC II expression) (Fig. S3A–C). The silencing procedure strongly reduced *CIITA* expression on the mRNA level in both cell lines upon KD (Fig. S3A,B). Furthermore, CIITA downregulation caused a decreased expression of the MHC II isotypes HLA‐DR‐A and HLA DP‐A in WM793 cells (Fig. S3B). Since 1205Lu does not express MHC II on the cell surface, we did not observe differences upon CIITA KD, whereas WM793 CIITA KD cells were expressing less MHC II on their cell surface (Fig. S3C). These results confirmed, also on a protein level, that CIITA regulates MHC II surface expression. Furthermore, we observed that CIITA knockdown in WM793 cells resulted in increased susceptibility to NKmK, whereas CIITA knockdown in 1205Lu cells did not alter susceptibility compared to control cells (Fig. S3D,E).

Since MHC II is known to interact with lymphocyte activation gene 3 (LAG3) [54], an immune checkpoint inhibitor which is also present on primary NK‐cells [55], we sought to examine the direct impact of MHC II and LAG‐3 interaction in melanoma resistance to NKmK. First, we tested LAG3 expression on primary NK‐cells‐ of two healthy donors (Fig. S3H). We detected LAG3 expression on NK‐cells, even though the population of LAG‐3‐positive cells was around 10% or below. Melanoma cells (control or treated with IFNγ) were used for cytotoxicity assays with primary NK‐cells, treated with an Fc block and incubated with either isotype control (ctrl ab) or a LAG3 blocking antibody (αLAG3), which was previously described to disturb the interaction between MHC II and LAG3 [54, 56]. Although we revealed MHC II expression on melanoma cells and LAG‐3 on NK‐cells, we could not detect any differences in NKmK by blocking LAG‐3. This was true for control as well as IFNγ‐treated melanoma cells (Fig. S3I,J).

Given that melanoma resistance to NK‐cell cytotoxicity is mediated by IFNγ (Fig. 4), we examined the IFNγ role in regulating MHC II expression on melanoma cells during co‐culture with NK‐cells. Blocking IFNγ activity during co‐culture reduced MHC II protein levels to those of untreated control cells, whereas cells treated with an isotype control antibody maintained elevated MHC II expression (Fig. 6F), indicating an IFNγ‐dependent mechanism. These results clearly indicated that direct interactions between melanoma and NK‐cells strongly affect gene expression and protein abundance in melanoma cells. This process is IFNγ‐dependent and involves the upregulation of MHC II, primarily mediated by its master transcriptional regulator, CIITA. Collectively, our findings highlight the importance of NK‐cell–melanoma interactions in shaping the tumor immune landscape and identify potential targets for enhancing NK‐cell‐based immunotherapies.

### 3.7 DMF influences resistance formation and protein expression in melanoma cells

Since we identified IFNγ to induce melanoma resistance formation to NKmK, we proceeded with pharmacological targeting of IFNγ signaling as a tool to reduce the NK‐cell‐induced immune evasion in melanoma cells. Specifically, we aimed to disturb the IFNγ‐induced CIITA and MHC II upregulation.

**Fig. 7:**
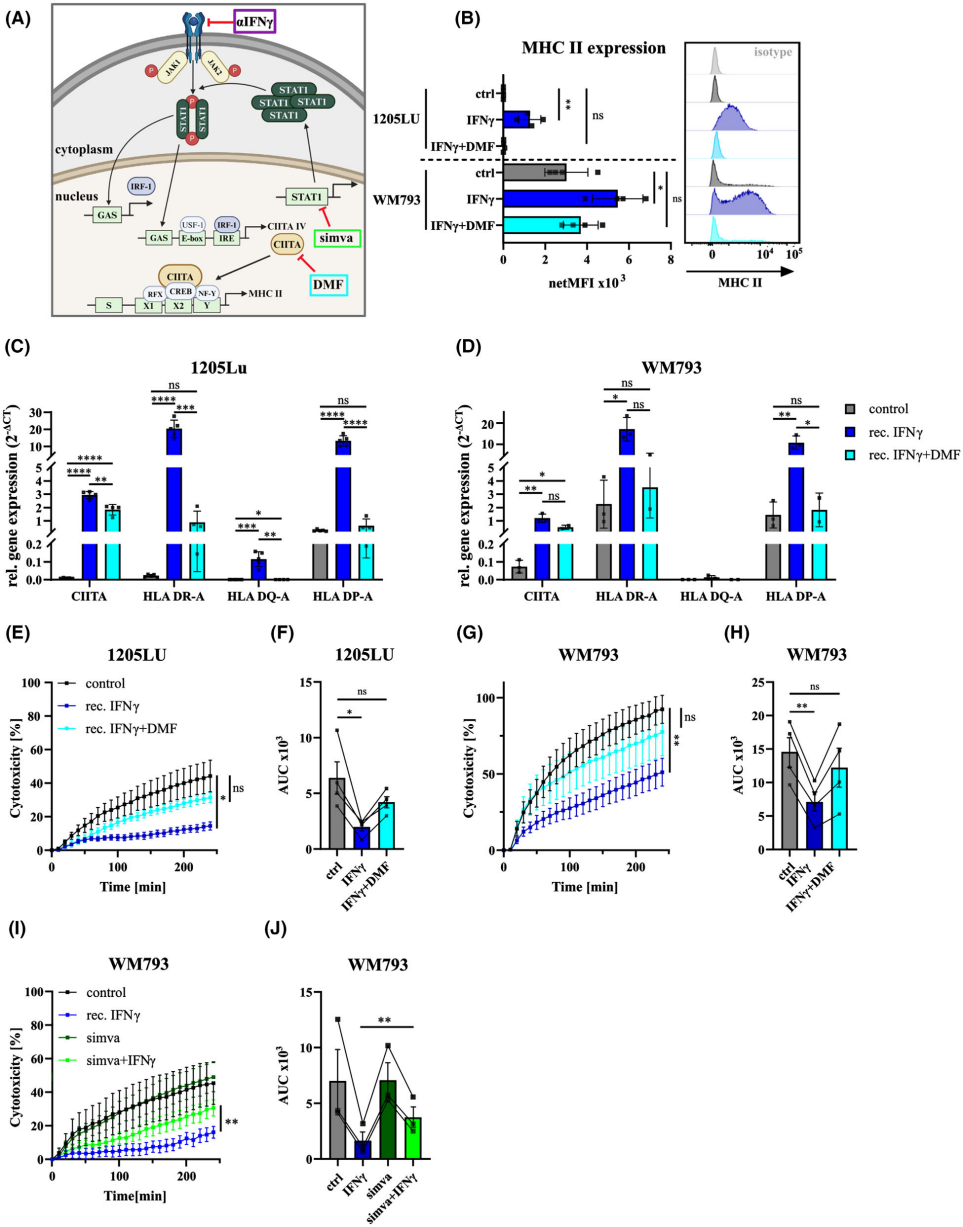
Dimethyl fumarate rescues melanoma resistance formation and MHC II expression after IFNγ stimulation. (A) Schematic of IFNγ‐induced MHC II expression with DMF and simvastatin as chemical compounds interfering with the depicted pathway. Adopted from [48, 57]. Image created with BioRender.com. (B) Flow cytometric analysis of MHC II expression of control cells (ctrl) or after treatment with recombinant IFNγ (rec.) or in combination with DMF (rec. IFNγ + DMF), *n* = 4, statistical analyses using two‐tailed unpaired Student's *t*‐test. (C, D) Gene expression analysis of MHC II isotypes and *CIITA* of (C) 1205Lu in control cells (*n* = 5), rec. IFNγ (*n* = 5) and rec.IFNγ + DMF (*n* = 4) and (D) WM793 in control cells (*n* = 3), rec. IFNγ (*n* = 3) and rec.IFNγ + DMF (*n* = 2). (E–H) Cytotoxicity assays of control, IFNγ‐treated (rec. IFNγ) or IFNγ and DMF‐treated cells (rec. IFNγ + DMF); (E, F) 1205Lu (*n* = 4), (E) kinetics of NKmK with (E) quantified AUC; each dot represents a single NK‐cell donor. (G, H) Cytotoxicity assay of WM793 (*n* = 4) with (F) Killing kinetics and (G) quantified AUC. (I, J) Cytotoxicity assay of WM793 control cells or treated with IFNγ or simvastatin (simva) alone or in combination; (I) killing kinetics (*n* = 3); and (J) quantified AUC, each dot represents a single donor (*n* = 3); data presented as mean ± SEM, statistical analyses using two‐tailed paired Student's *t*‐test of AUCs, (ns) *P* > 0.05; (*) *P* < 0.05; (**) *P* < 0.01; (***) *P* < 0.001; (****) *P* < 0.0001.

IFNγ binding to its receptor triggers phosphorylation, recruiting and activating JAK1 and JAK2, which then phosphorylate STAT1. Phosphorylated STAT1 forms homodimers (GAF) that translocate to the nucleus and bind the γ‐activation sequence (GAS), inducing IRF1 and IRF2 expression. IRF1/2 and GAF bind promoter IV of CIITA, driving its expression. In the presence of an enhanceosome complex, CIITA initiates MHC II expression. In order to target this signaling cascade, we used two drug compounds, namely dimethyl fumarate (DMF) and simvastatin, both shown to interfere with IFNγ signaling (Fig. 7A and [48, 57, 58]).

We treated 1205Lu and WM793 cells with recombinant IFNγ, alone or combined with DMF, for 24 h and found that DMF blocked the effect of IFNγ, mirroring the results observed with the IFNγ‐blocking antibody during co‐culture. Using DMF treatment, MHC II protein expression recovered back to control levels in both 1205Lu and WM793 compared to IFNγ treated cells (Fig. 7B). RT‐qPCR analysis revealed decreased gene expression of several MHC II isotypes after DMF treatment compared to IFNγ alone in 1205Lu and WM793 (Fig. 7C,D). Gene expression of *CIITA* was only diminished in 1205Lu compared to IFNγ alone indicating that DMF might inhibit CIITA activity rather than its gene expression. Notably, not only MHC II expression was restored, but DMF treatment also prevented the melanoma resistant phenotype, leading to increased NKmK susceptibility (Fig. 7E–H).

To test if DMF treatment is IFNγ‐specific, we determined MHC II expression and NKmK in melanoma cells that were not treated with IFNγ or exposed to NK‐cell co‐culture. As demonstrated, DMF did not affect MHC II expression nor the susceptibility to NKmK (Fig. S4A,B). Further, we analyzed if DMF could rescue early steps in the IFNγ‐initiated signaling cascade leading to MHC II upregulation. IFNγ‐induced *Usf1*, *Irf1*, and *Irf2* expression was not affected by DMF (Fig. S4C–E), suggesting that DMF directly inhibits CIITA activity and has no other unspecific influence on the IFNγ‐induced signaling.

Simvastatin, a 3‐hydroxy‐3‐methylglutaryl coenzyme A (HMG‐CoA) reductase inhibitor, is widely used as a cholesterol‐lowering drug. Previous studies have shown that simvastatin can inhibit IFNγ‐induced MHC II expression through a CIITA‐dependent mechanism [57, 59]. This led us to hypothesize that, similar to DMF, simvastatin might also reduce melanoma resistance formation.

To test this, we pretreated melanoma cells with IFNγ alone or in combination with simvastatin (10 μm). Our results clearly demonstrated that, like DMF, simvastatin effectively blocks IFNγ‐induced melanoma cell resistance to NKmK (Fig. 7I,J).

These findings underscore the critical role of the IFNγ‐induced CIITA pathway in melanoma immune resistance. By targeting this pathway with DMF and simvastatin, we highlight the potential of pharmacological interventions to sensitize melanoma cells to NK‐cell cytotoxicity. This approach could pave the way for combinatorial strategies that enhance the efficacy of NK‐cell‐based immunotherapies and improve treatment outcomes for melanoma patients.

## 4 Discussion

Melanoma is a heterogeneous cancer with a high mutational load and metastatic propensity, exhibiting distinct features between primary cancers and metastases [60, 61, 62]. Targeted therapies suppress key pathways like RAS–RAF–MEK–ERK [63], and immune checkpoint inhibitors against PD‐1 and CTLA‐4 have shown efficacy in treating advanced melanoma but also other cancers [64, 65, 66, 67]. Despite this, resistance and low response rates remain as significant challenges in treating melanoma [4].

Phenotypic plasticity influences melanoma aggressiveness, therapy response, and disease outcomes [68, 69]. The tumor microenvironment, including immune cell infiltration, drives melanoma phenotype switching via secreted factors such as cytokines and ROS [18, 70, 71, 72, 73]. CTLs and NK‐cells interact to regulate melanoma susceptibility to immune cytotoxicity [74], but the role of NK‐cells in phenotype switching and therapeutic sensitivity is less understood compared to the role of CTLs. Given the emerging potential of NK‐cell‐based therapies, understanding the NK‐melanoma cell interactions is critical. Previous studies demonstrated that NK‐cells not only induce melanoma cell cytotoxicity but also promote NK‐cell exhaustion and/or reduced melanoma sensitivity to immune‐based therapies [17, 19, 75]. However, these studies did not consider the role of different melanoma subtypes. Thus, evaluating NKmK in both less aggressive vertical growth phase melanoma cells and aggressive metastasis‐derived cell lines was essential.

In this study, we applied *2D* and *3D* NK‐melanoma cell co‐culture models to evaluate NKmK in four melanoma lines. By optimizing the co‐culture conditions, we identified key parameters governing these interactions. Importantly, we demonstrated that melanoma cells developed rapid resistance to NKmK following NK‐cell exposure. While NKmK varied depending on the cell line [25], resistance could be observed consistently in all cell lines and in both co‐culture models.

### 4.1 IFNγ

Single‐cell RNA sequencing has become a powerful tool for investigating cell‐specific responses in various cancers, including melanoma, offering detailed insights into tumor‐immune cell interactions [33, 34]. Utilizing this approach, we analyzed publicly available scRNAseq data from 15 melanoma patients with cutaneous, acral, brain, and lymph node metastases [33, 34] to identify key mediators of NK‐cell–melanoma cell interactions (Fig. 2). Among the top candidates, IFNγ emerged as a central factor, which we experimentally confirmed to be secreted in our co‐culture system. Furthermore, our data revealed that IFNγ release correlates with the susceptibility of control melanoma cells to NKmK. The important role of IFNγ in the context of cancer is evident [76] but also complex and not fully understood. In melanoma, IFNγ can lead to an epithelial to mesenchymal transition and to a transformation towards an undifferentiated and more invasive melanoma phenotype [19]. Further, there are studies underlining the effects of IFNγ on immune cell recognition, whereby IFNγ was demonstrated to increase MHC I expression in melanoma, inhibiting NK‐cell subsets in their melanoma clearance [20]. On the other hand, there are studies demonstrating a rather opposite effect, whereby IFNγ leads to a melanoma phenotype switch to a non‐aggressive, less metastasizing phenotype [77].

To examine the impact of IFNγ on melanoma gene expression, particularly on NK‐cell ligands, we analyzed RNA‐sequencing data from Grasso et al., where melanoma cells were treated with recombinant IFNγ. Consistent with previous studies, MHC I molecules were upregulated following IFNγ exposure [17, 20, 78]. Since the role of MHC I was already extensively studied in NKmK and given that in our previous study we did not observe a clear correlation between NKmK and MHC I expression [25], we focused on identifying other factors that affect NKmK in an IFNγ‐dependent manner. Notably, we found that a majority of IFNγ‐regulated ligands are MHC II‐related genes and that both NK‐cell co‐culture as well as IFNγ treatment lead to an upregulated gene expression of *HLA‐DP*, *‐DQ*, *‐DR*, and *CIITA*, with increased protein surface expression of MHC II. These findings were in line with previous publications which showed that IFNγ can regulate MHC II in several cell types including melanoma [79, 80, 81].

### 4.2 MHC II

Considering the melanoma phenotype classification defined by Mendez et al. [80], we categorized our melanoma cell lines based on their MHC II expression as follows: 1205Lu‐phenotype 2 (inducible MHC II), WM1366‐phenotype 3 (partial constitutive expression), and WM793‐ and WM3734a‐phenotype 4 (constitutive MHC II, enhanced by IFNγ). These phenotypes, covering more than 70% of all melanoma cases, all displayed IFNγ‐induced MHC II upregulation and developed resistance to NKmK upon IFNγ exposure, thus highlighting the relevance of MHC II in NKmK.

The role of MHC II in melanoma was widely discussed but many questions remain unsolved. Whereas healthy melanocytes do not bear MHC II, 20% of primary and 50% of metastatic melanoma lesions do express MHC II on their surface [82, 83]. In this context, MHC II expression is associated with metastasis and poor disease outcome [84, 85, 86]. On the other hand, a positive disease outcome was discussed with melanoma expressing MHC II [80, 87]. Apart from the effects on the general disease outcome, various effects on the immune system, and more specifically on NK‐cells, have been reported. While some studies suggest a negative effect of MHC II on NK‐cell cytotoxicity [88, 89], others report either an activating effect or no impact on NK‐cell functions [55]. Given these conflicting findings and the highly dynamic tumor microenvironment (TME)‐sensitive nature of MHC II expression, we aimed to conduct a more detailed and controlled analysis to better understand the role of MHC II in NKmK.

MHC II upregulation following co‐culture and IFNγ treatment suggested a potential inhibitory effect on NK‐cells. While MHC II typically interacts with CD4 on T cells, it can also bind LAG‐3, an immune checkpoint receptor [90]. Although LAG‐3 is known to inhibit T cells, its role in NK‐cells remains controversial, with some studies reporting inhibitory effects [91], while others report no impact on NK‐cell cytotoxicity [92]. In patients treated with anti‐LAG‐3 and anti‐PD‐1 therapy, effects on NK‐cells, regulatory T cells, and CTLs were observed [93]. Using a LAG3 blocking antibody (17B4) [56], we found no impact on NKmK, supporting previous *in vitro* data [92, 94]. The discrepancy between *in vitro* and *in vivo* observations might be explained by other interactions of LAG‐3, for instance with L‐section and FGL1 [95]. Nevertheless, the potential contribution of MHC II upregulation as a molecular mechanism underlying melanoma cell resistance to NK‐cell cytotoxicity remains likely.

### 4.3 CIITA

CIITA is a non‐DNA‐binding co‐activator that plays a critical role in the regulation of MHC II [96]. Beyond its role in the regulation of MHC II, CIITA can regulate some non‐MHC genes and appears to have a broader impact on gene expression [97]. Using siRNA targeting CIITA, we confirmed the regulation of MHC II by CIITA on gene expression and protein surface abundance. Knowing the influence of CIITA on MHC II, we further inhibited CIITA activity using DMF and simvastatin. DMF, an FDA‐approved drug for autoimmune diseases [26, 27], was previously shown to block IFNγ‐induced CIITA expression [58]. In our study, DMF reversed melanoma resistance to NKmK, reducing MHC II expression without affecting upstream IFNγ targets (USF1, IRF1, IRF2). Interestingly, a previous study demonstrated that in MS patients, the number of NK‐cells in the peripheral blood increased during DMF treatment, while CTL counts decreased [98]. Furthermore, the main metabolite of DMF, monomethyl fumarate (MMF), has been shown to increase NK‐cell cytotoxicity against tumor cells, which is associated with an upregulation of the activating receptor NKp46 [99, 100].

Similarly, simvastatin, a widely used HMG‐CoA reductase inhibitor [59], reduced melanoma resistance by inhibiting CIITA expression through STAT1 modulation [57]. Although simvastatin can suppress NK‐cell activity [101], its application in a controlled manner could improve melanoma sensitivity to NK‐cell‐based therapies.

## 5 Conclusions

Our current study enhances the understanding of melanoma cell resistance to NK‐cell cytotoxicity by demonstrating that resistance to NKmK is induced by IFNγ‐mediated interactions between melanoma and NK‐cells, with IFNγ, CIITA, and MHC II playing central roles. Furthermore, we reveal that drugs such as DMF and simvastatin can mitigate this resistance by inhibiting CIITA and, hence, MHC II expression. These findings provide valuable insights into the molecular mechanisms underlying melanoma resistance and suggest potential therapeutic strategies that warrant further investigation.

## Conflict of interest

The authors declare no conflict of interest.

## Author contributions

LCMK, SC, and IB conceived and designed the project; LCMK, R‐MK, CI, JL, and JF acquired and analyzed the data; LCMK, CI, R‐MK, SC, and IB interpreted the data; LCMK, CI, and IB acquired funding; LCMK, SC, and IB supervised the project; LCMK and IB wrote the paper.

## Peer review

The peer review history for this article is available at https://www.webofscience.com/api/gateway/wos/peer‐review/10.1002/1878‐0261.70133.

## Supporting information


**Fig. S1.** Resistant melanoma phenotype after NK‐cell co‐culture recovers over time.
**Fig. S2.** Differential melanoma gene expression.
**Fig. S3.** Increase of melanoma susceptibility to NKmK by knock‐down of CIITA but not by blocking of LAG‐3 and MHC II interactions.
**Fig. S4.** Effects of DMF treatment on NKmK, MHC II protein expression and IFNγ pathway regulation.

## Data Availability

The data presented in this study are available upon request from the corresponding author.
