# Supplementary material for: The IFNγ‐CIITA‐MHC II axis modulates melanoma cell susceptibility to NK‐cell‐mediated cytotoxicity
Source: Mol Oncol. 2025 Oct 13;19(11):3096–119. doi: 10.1002/1878-0261.70133 (PMC12591318; doi:10.1002/1878-0261.70133)
Supplement: Supplementary file 1 — Fig. S1. Resistant melanoma phenotype after NK‐cell co‐culture recovers over time. Fig. S2. Differential melanoma gene expression. Fig. S3. Increase of melanoma susceptibility to NKmK by knock‐down of CIITA but not by blocking of LAG‐3 and MHC II interactions. Fig. S4. Effects of DMF treatment on NKmK, MHC II protein expression and IFNγ pathway regulation. [file MOL2-19-3096-s001.zip › mol270133-sup-0001-supinfo.docx]

Supplementary Figure Legends

Figure S1: Resistant melanoma phenotype after NK‑cell‑co-culture recovers over time. Flow cytometric characterisation of A-D) PBMCs and E-H) isolated NK-cells. NK cell defining population (CD3- and CH56+) in D) PBMCs 6%, in H) isolated NK cells 97%. A-H) are representative data of one donor, displaying 20,000 events. I) Schematic of co-culture-based model with recovery. Created with BioRender.com. Relative end point lysis of control (ctrl), co-cultured (cc) or melanoma cells previously co-cultured and recovered for 24, 48, or 72 h (rec) without NK-cells present in culture. J) 1205Lu (n=3) and K) WM793 (cc and 24 h rec: n=7; 48 h and 72 h rec: n=3), each point represents a single NK-cell donor; data normalised to the mean of the control, data presented as mean ± SD (*)p<0.05; (**)p<0.01; (***)p<0.001; (****)p<0.0001; assessed by two-tailed paired Student’s *t*-test compared to control.

Figure S2: Differential melanoma gene expression. A-C) Differential gene expression in the immune cell landscape within the melanoma tissue of 15 melanoma samples (14,661 cells) of A) IFNGR1/2, B) MHC I-related genes (namely: HLA-A, -B, -C,-E, -F, -G) and C) MHC II-related genes (all identified genes). Data obtained from [33, 34]. D-G) Gene expression of NK-cell ligands in 1205Lu and WM793 control (grey) or IFNγ-treated (blue) cells. D) *Ulbp2* (1205Lu: n=3; WM793: n=4), E) *Ulbp3* (n=3), F) *Ceacam1* (1205Lu: n=3; WM793: n=4) and G) *Lgals9* (n=3). H-I) Validation of CEACAM1 as a possible regulator of melanoma resistance formation. H) *Ceacam1* gene expression in 1205Lu and WM793 after transfection using siRNA control (siCtrl) or targeting *Ceacam1* (siCEACAM1) (1205Lu: n=1 biological replicate; n=3 technical replicates, WM793: n=2 biological replicates; n=5 technical replicates) and I) their subsequent susceptibility to NKmK (1205Lu: n=3, WM793: n=4). Samples were either not treated (wo) or treated with IFNγ (IFNγ). Data presented as mean ± SD. Statistical analysis by two-tailed unpaired Student’s t-test, (ns)p>0.05; (*)p<0.05; (**)p<0.01; (***)p<0.001; (****)p<0.0001.

Figure S3: Increase of melanoma susceptibility to NKmK by knock-down of CIITA but not by blocking of LAG-3 and MHC II interactions. Knock-down (KD) of *CIITA* expression (siCIITA) using siRNA; KD quantification using qRT-PCR in A) 1205Lu with 33% expression of *CIITA* compared to siCtrl (n=1 biological replicate; n=2 technical replicates) and B) in WM793 with 28% *CIITA* expression (n=1 biological replicate; n=2 technical replicates). CIITA influences MHC II A-B) gene expression and C) protein surface expression in 1205Lu and WM793, displaying n=10,000 events. Cytotoxicity assays of control (siCtrl) and *CIITA* KD cells (siCIITA) of D-E) 1205Lu (n=4) and F-G) WM793 (n=4) depicted with killing kinetics and area under the curve (AUC). H) LAG-3 expression of primary NK-cells of two donors as percent of live cells; histograms demonstrate gating of the LAG-3 positive population, n=10,000 events displayed. I-J) Cytotoxicity assay of control and recombinant IFNγ- treated cells (IFNγ) treated with anti-LAG3 antibody (αLAG3) or respective isotype control antibody (ctrl ab). I) 1205Lu (n=2) J) WM793 (n=2) using donors depicted in H), data are presented as mean ±SEM. Statistical analysis by two-tailed paired Student’s t-test, (ns) not significant; (**)p<0.01

Figure S4: Effects of DMF treatment on NKmK, MHC II protein expression and IFNγ pathway regulation. A) Flow cytometric analysis of MHC II expression of control (ctrl) melanoma cells or after treatment with DMF in 1205Lu (n=3) and WM793 (n=4). B) Cytotoxicity of control and DMF-treated WM793 (n=2), data presented as mean ±SEM. C-E) Gene expression analysis of C) *Usf1* (n=3), D) *Irf1* (n=3), and E) *Irf2* (n=3) after treatment with rec. IFNγ alone or in combination with DMF. Statistical analysis by two-tailed unpaired Student’s t-test, (ns) not significant; (*)p<0.05; (**)p<0.01; (***)p<0.001; (****)p<0.0001.
