# Supplementary figures and images for: The IFNγ‐CIITA‐MHC II axis modulates melanoma cell susceptibility to NK‐cell‐mediated cytotoxicity
Source: Mol Oncol. 2025 Oct 13;19(11):3096–119. doi: 10.1002/1878-0261.70133 (PMC12591318; doi:10.1002/1878-0261.70133)

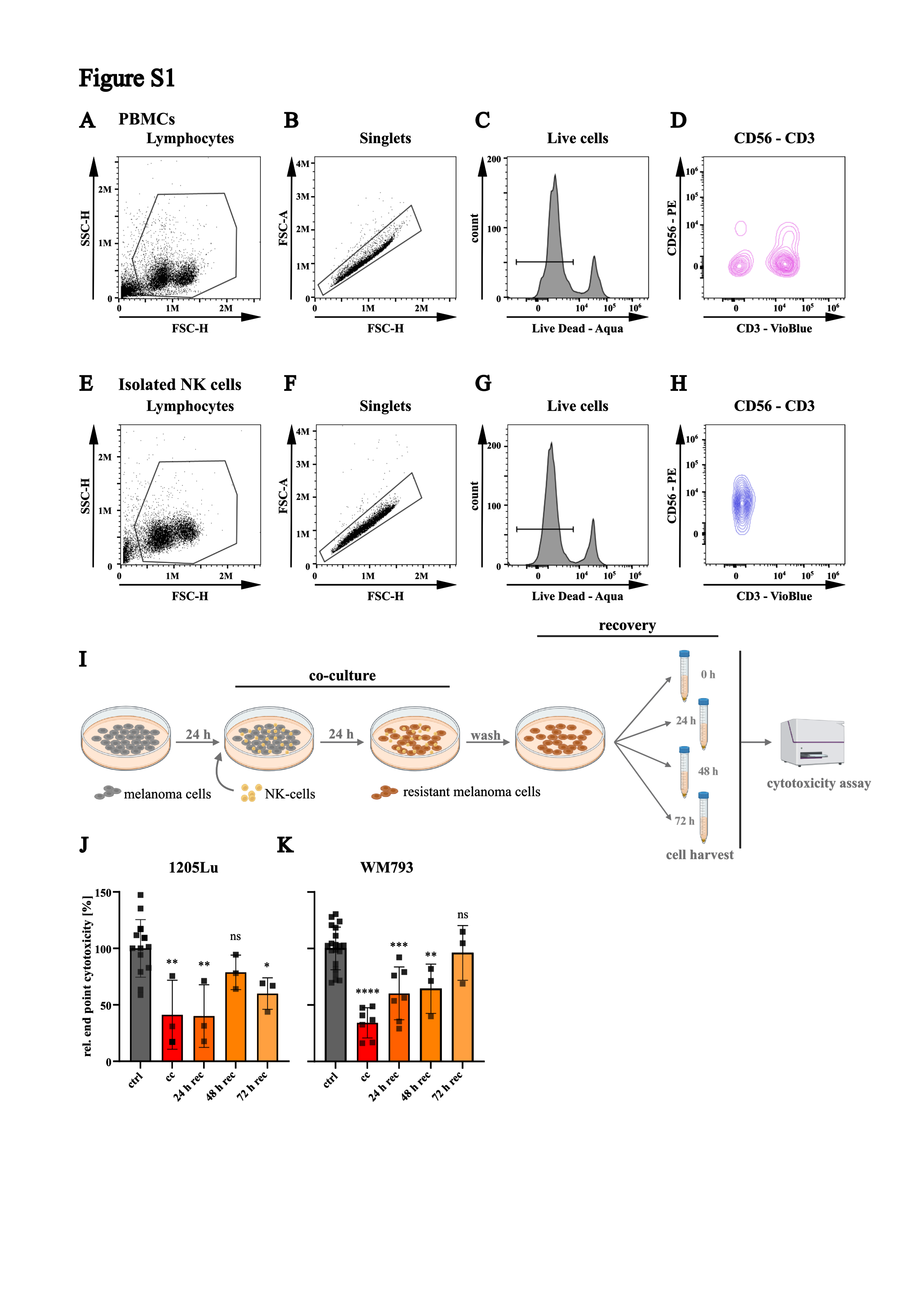

Supplement: Supplementary file 1 — Fig. S1. Resistant melanoma phenotype after NK‐cell co‐culture recovers over time. Fig. S2. Differential melanoma gene expression. Fig. S3. Increase of melanoma susceptibility to NKmK by knock‐down of CIITA but not by blocking of LAG‐3 and MHC II interactions. Fig. S4. Effects of DMF treatment on NKmK, MHC II protein expression and IFNγ pathway regulation. [file MOL2-19-3096-s001.zip › mol270133-sup-0002-FigureS1.tiff]

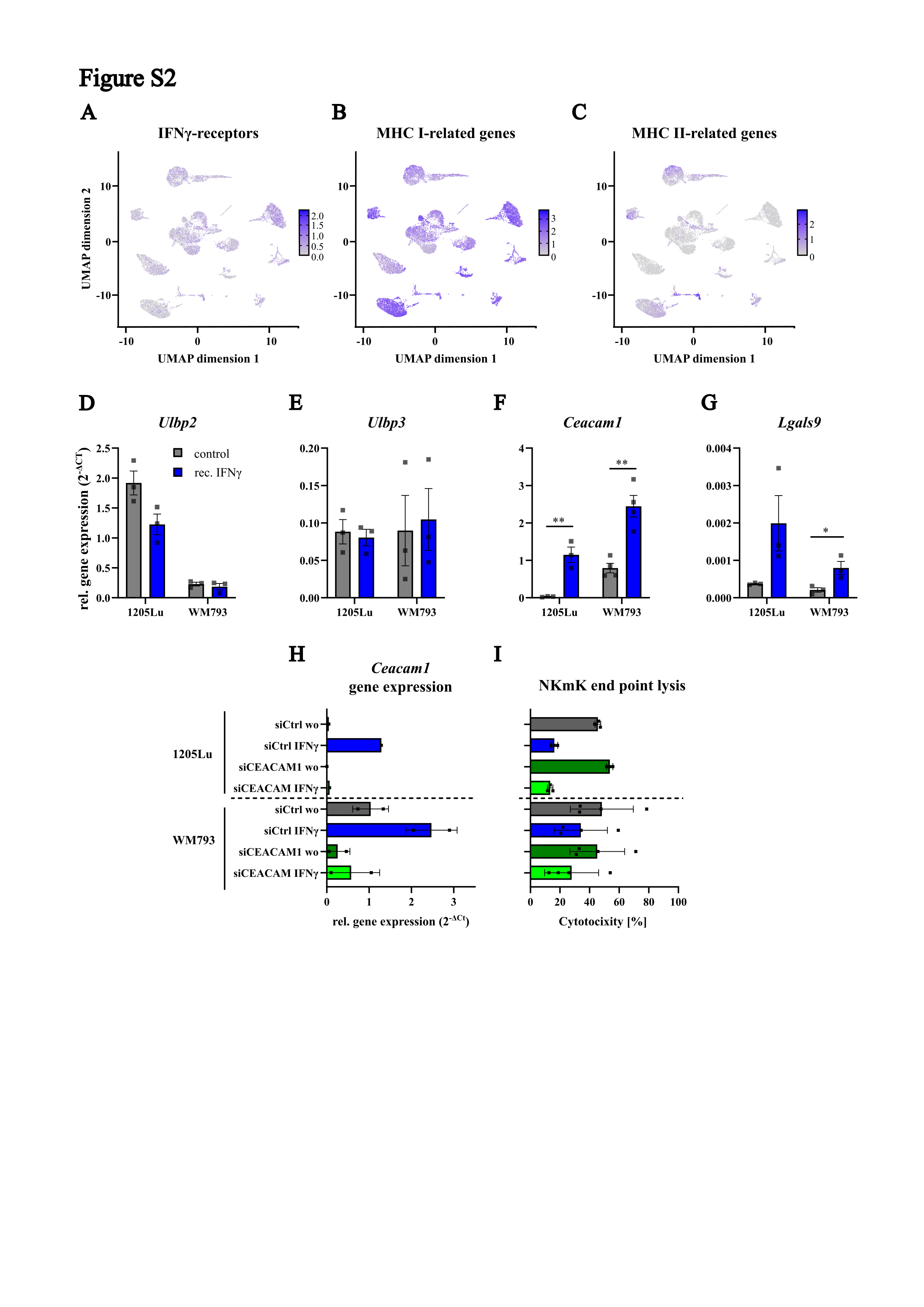

Supplement: Supplementary file 1 — Fig. S1. Resistant melanoma phenotype after NK‐cell co‐culture recovers over time. Fig. S2. Differential melanoma gene expression. Fig. S3. Increase of melanoma susceptibility to NKmK by knock‐down of CIITA but not by blocking of LAG‐3 and MHC II interactions. Fig. S4. Effects of DMF treatment on NKmK, MHC II protein expression and IFNγ pathway regulation. [file MOL2-19-3096-s001.zip › mol270133-sup-0003-FigureS2.tiff]

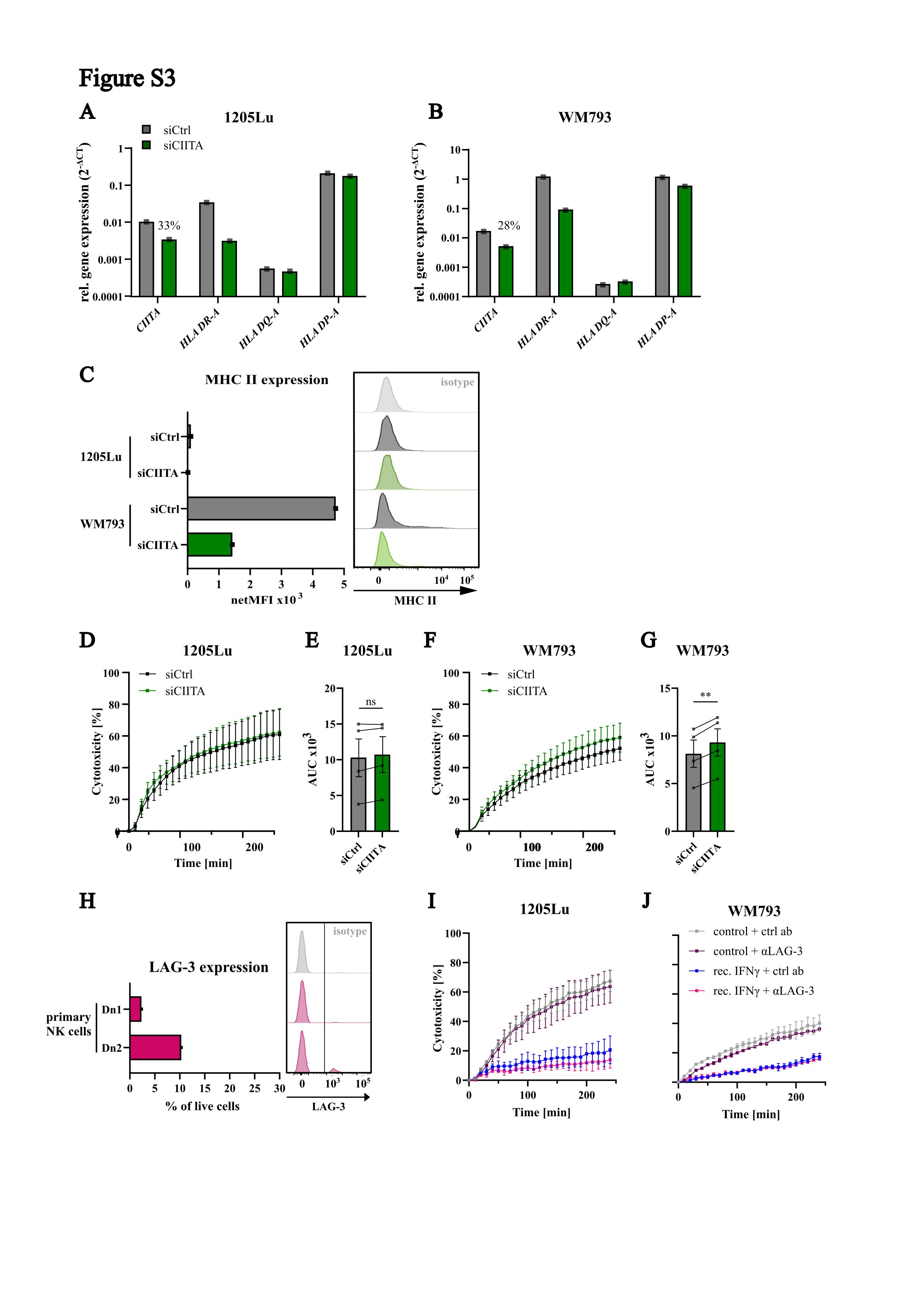

Supplement: Supplementary file 1 — Fig. S1. Resistant melanoma phenotype after NK‐cell co‐culture recovers over time. Fig. S2. Differential melanoma gene expression. Fig. S3. Increase of melanoma susceptibility to NKmK by knock‐down of CIITA but not by blocking of LAG‐3 and MHC II interactions. Fig. S4. Effects of DMF treatment on NKmK, MHC II protein expression and IFNγ pathway regulation. [file MOL2-19-3096-s001.zip › mol270133-sup-0004-FigureS3.tiff]

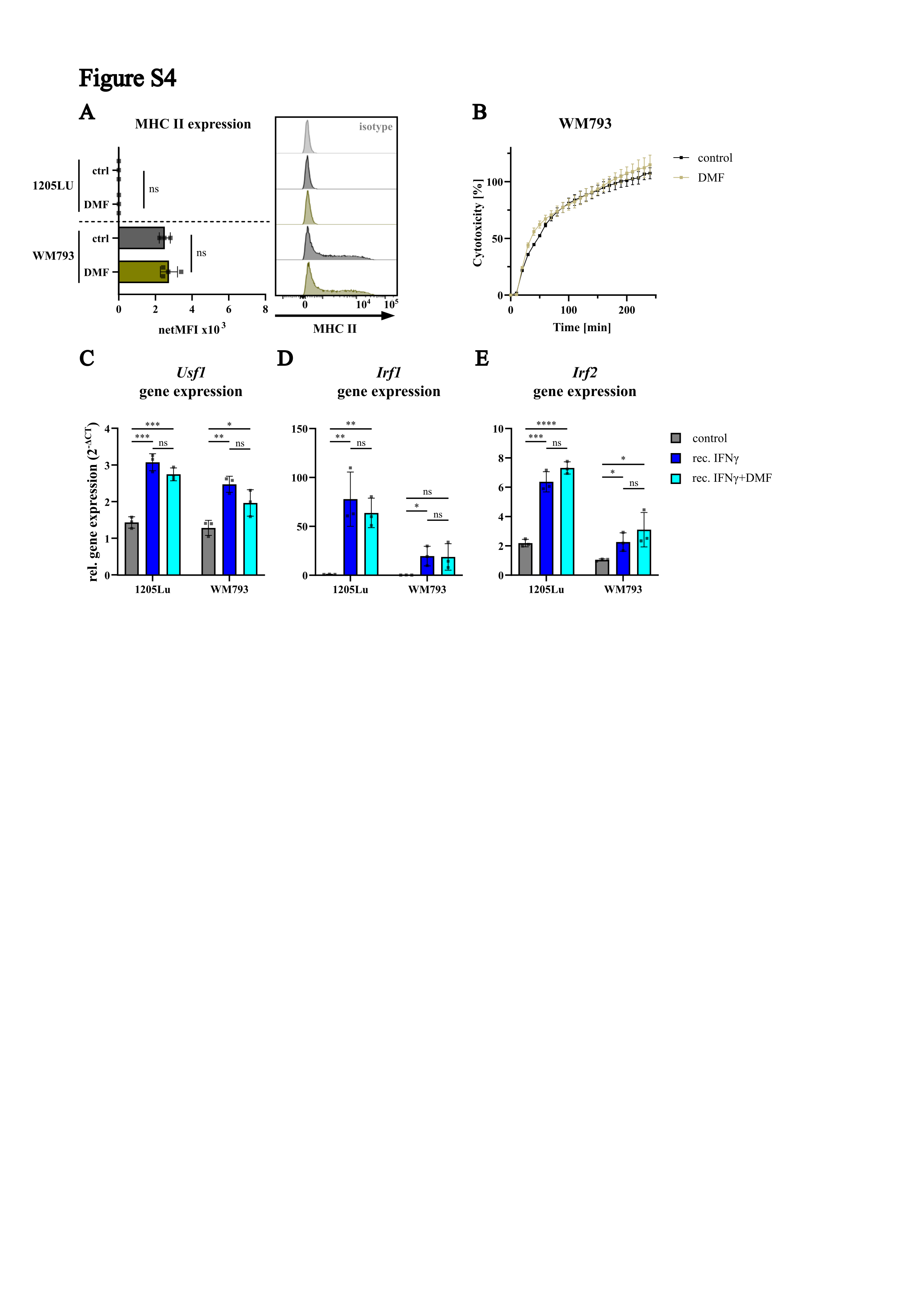

Supplement: Supplementary file 1 — Fig. S1. Resistant melanoma phenotype after NK‐cell co‐culture recovers over time. Fig. S2. Differential melanoma gene expression. Fig. S3. Increase of melanoma susceptibility to NKmK by knock‐down of CIITA but not by blocking of LAG‐3 and MHC II interactions. Fig. S4. Effects of DMF treatment on NKmK, MHC II protein expression and IFNγ pathway regulation. [file MOL2-19-3096-s001.zip › mol270133-sup-0005-FigureS4.tiff]
